# Integrative single-cell analysis of cardiac and pulmonary sarcoidosis using publicly available cardiac and bronchoalveolar lavage fluid sequencing datasets

**DOI:** 10.3389/fcvm.2023.1227818

**Published:** 2023-07-28

**Authors:** Abdel Daoud, Diego A. Lema, Taejoon Won, Daniela Čiháková

**Affiliations:** ^1^ W. Harry Feinstone Department of Molecular Microbiology and Immunology, Johns Hopkins University Bloomberg School of Public Health, Baltimore, MD, United States; ^2^ Department of Pathology, Johns Hopkins University School of Medicine, Baltimore, MD, United States

**Keywords:** cardiac sarcoidosis (CS), pulmonary sarcoidosis, single-cell RNA-seq (scRNA-seq), single-nucleus RNA-seq, meta-analysis, bioinformactics

## Abstract

**Introduction:**

Cardiac presentation of autoimmune sarcoidosis, known as cardiac sarcoidosis (CS), is a poorly understood disease with high mortality and low diagnosis rate. While CS is an immunological syndrome, little is known about how cardiac parenchymal and stromal cells mediate its pathogenesis. Moreover, while most current sarcoidosis research is based on research in pulmonary sarcoidosis (PS), it remains unclear how much both presentations of sarcoidosis overlap. To tackle these concerns, we leveraged publicly available sarcoidosis transcriptomic datasets.

**Methods:**

Two publicly available bronchoalveolar lavage single-cell RNA sequencing datasets were integrated to analyze PS relative to control. Additionally, two publicly available cardiac single-nucleus RNA sequencing datasets were integrated to analyze CS relative to control. Following integration, we ran cell-cell communication, transcription factor, and differential expression analyses on parenchymal, stromal, and immune subsets identified in our analysis.

**Results:**

Our analysis revealed that there was an expansion of stromal and immune cells in PS and CS. We also observed upregulation of Th17.1 and attenuated activation transcriptional profiles in the immune cells of CS and PS relative to control. Additionally, we found upregulation of pro-inflammatory and pro-fibrotic transcriptional profiles in the cardiac stromal cells of CS relative to control. We also found that cardiomyocytes exhibited upregulated cardiac stress and proliferation transcriptional profiles in CS relative to control.

**Conclusions:**

Our integrative transcriptomic analysis shows that despite tissue-specific differences, there are shared transcriptional trends between CS and PS. It also shows that stromal and parenchymal populations exhibit transcriptional trends that could explain their pathogenic role in CS.

## Introduction

Sarcoidosis is a multisystemic auto-inflammatory syndrome marked by the formation of non-necrotizing granulomas which are defined as small immune cellular aggregates constituted mainly of multinucleated macrophage giant cells as well as CD4^+^ and CD8^+^ T lymphocytes and surrounded by epithelial and fibrotic layers (1). While pulmonary sarcoidosis (PS), characterized by lung and/or intrathoracic lymph node involvement, constitutes at least 90% of sarcoidosis cases (2), only 5% of sarcoidosis patients present with clinically overt cardiac sarcoidosis (CS), defined as sarcoidosis with myocardial involvement (3, 4). It is estimated that at least a third of sarcoidosis cases have subclinical CS (4). This is further exacerbated by the fact that at least half CS cases present in an isolated manner, as opposed to a multisystem presentation, with sudden cardiac death constituting the first clinical sign of most of such cases (4). As sarcoidosis is an intricate and poorly understood immunological syndrome (1), many techniques have been utilized to better understand its pathobiology. Single cell resolution RNA sequencing techniques, such as single-cell RNA-sequencing (scRNA-Seq) and single-nucleus RNA-sequencing (snRNA-Seq), are examples of such techniques. Both scRNA-Seq and snRNA-Seq are particularly well-suited to understanding the transcriptional mechanism of multi-agent syndromes, such as sarcoidosis. While bulk RNA-sequencing has been utilized to study sarcoidosis, namely PS, in numerous past studies (5–7), there are very few scRNA-Seq studies that investigate sarcoidotic syndromes. Even PS, the most canonical of such syndromes, has only been investigated once so far via scRNA-Seq in a bronchoalveolar lavage fluid (BAL)-focused study (8). This is problematic because of the myeloid bias of BAL samples as well as the fact that this study failed to truly utilize the potential of the scRNA-Seq technology, including the ability to run cell-cell communication and protein-protein interaction analyses. Recently, a snRNA-Seq study performed transcriptional profiling of cardiac macrophages in CS (9). However, that study lacked a healthy control comparison and was in general more focused on myeloid populations involved in CS. In this study, we attempted to integrate publicly available scRNA-Seq and snRNA-Seq CS and PS datasets with appropriate controls utilizing novel transcriptomic algorithms, such as cell-cell communication and transcription factor analysis workflows. This approach allowed us to transcriptionally profile BAL and cardiac immune cells involved in sarcoidosis pathology, namely macrophages and T cells, as well as cardiac stromal cells implicated in CS, such as cardiac fibroblasts and endothelial cells. Using this approach, we show that, regardless of the tissue of presentation, sarcoidotic macrophages and T cells upregulate activation attenuation transcriptional profiles relative to control. We also show that CS cardiac fibroblasts and endothelial cells are enriched for pro-inflammatory and pro-fibrotic pathways relative to control. In addition, we show that viable cardiomyocytes in CS exhibit upregulated pro-inflammatory and cardiac stress transcriptional pathways relative to control.

## Material and methods

### Publicly available dataset and patient characterization

Two publicly available scRNA-Seq datasets were utilized to investigate PS, a control BAL dataset as well as a PS BAL dataset. The control scRNA-Seq dataset was retrieved using the accession code: GSE193782 (10). This dataset included 4 healthy control patients and 3 cystic fibrosis patients. Only the healthy control sequencing data were retained for the PS analysis. The PS scRNA-Seq dataset was retrieved using the accession code: GSE184735 (11). This dataset included 4 PS patients, 3 chronic beryllium disease patients and 2 beryllium-sensitized patients. Only the PS patients were retained for the PS analysis. The available clinical characteristics of the patients utilized for the PS analysis are shown in Supplementary Table S1 (10, 11).

Two publicly available snRNA-Seq were utilized to investigate CS, a control cardiac dataset as well as a CS cardiac dataset. The control snRNA-Seq dataset was retrieved using the accession code: ERP123138 (12). This dataset included sequencing data of 14 healthy control patients collected from various regions of the heart. The CS snRNA-Seq dataset was retrieved using the accession code: GSE205734 (9). This dataset included sequencing data of 4 CS patients and 3 ischemic cardiomyopathy patients collected from the apex of the heart. Only the CS patients were retained for the CS analysis. The available clinical characteristics of the patients utilized for the CS analysis are shown in Supplementary Table S2 (9, 12).

### PS scRNA-Seq workflow

The Seurat scRNA-Seq SCT integration and analysis workflow was utilized (13). Separate Seurat objects were created for each of the PS samples (*n* = 4) using the Read10X() function after which they were merged into one Seurat object using the merge() function. For the healthy samples (*n* = 4), counts were read in using the Read10X() function after which they were merged into one Seurat object. To normalize for sample size, the control samples were downsampled to 8,000 cells. For the quality control of the healthy samples, cells with nFeature_RNA less than 1,850 or more than 7,450, and nCount_RNA more than 85,000 as well as mitochondrial gene content higher than 12% were discarded from further analysis. For the quality control of the PS samples, cells with nFeature_RNA more than 7,100 or less than 2,450, and nCount_RNA more than 87,750 as well as mitochondrial gene content higher than 15% were discarded from further analysis. The healthy and PS Seurat objects were separately normalized via the regularized negative binomial regression method by running the SCTransform() function to obtain SCT count assays, which were utilized for integration. Uniform Manifold Approximation and Projection (UMAP) reduction was run using the first 45 batch corrected principal component analysis (PCA) component. PCA batch correction was done using the Harmony package (14).

For subsequent gene expression analysis, the default assay was reverted to the RNA assay which was consequently log-normalized. Expression of canonical BAL cell type markers (Supplementary Table S3) was utilized to provide biological cluster annotations.

### CS snRNA-Seq workflow

The Seurat snRNA-Seq SCT integration and analysis workflow was utilized (13). Separate Seurat objects were created for each of the CS samples (*n* = 4) using the Read10X() function after which they were merged into one Seurat object using the merge() function. For the healthy samples (*n* = 14), counts were read in and filtered for those in the apex region using the provided metadata and were merged into one Seurat object. The CS dataset yielded approximately 26,000 nuclei. To normalize for sample size, the control samples were downsampled to 26,000 nuclei. For the quality control of the healthy samples, nuclei with nFeature_RNA less than 450 or more than 3,400, and nCount_RNA more than 7,250 were discarded from further analysis. For the quality control of the CS samples, nuclei with nFeature_RNA less than 520 or more than 2,800, and nCount_RNA more than 7,600 were discarded from further analysis. As cardiomyocytes tend to have a high mitochondrial gene content, the use of mitochondrial gene content as a quality control metric was postponed till biological cluster annotation. The healthy and CS Seurat object were separately normalized via the regularized negative binomial regression method by running the SCTransform() function to obtain SCT count assays, which were utilized for integration. UMAP reduction was run using the first 50 batch corrected PCA component. PCA batch correction was done using the Harmony package (14).

For subsequent gene expression analysis, the default assay was reverted to the RNA assay which was consequently log-normalized. Expression of canonical cardiac cell type markers (Supplementary Table S4) was utilized to provide biological cluster annotations. Afterwards, the biological labels were exported, and the complete workflow was repeated with the added step that mitochondrial gene content was used as a quality control metric for non-cardiomyocyte cells. For the healthy sample, non-cardiomyocyte cells with a mitochondrial gene content greater than 5% were excluded. For the CS sample, non-cardiomyocyte cells with a mitochondrial gene content greater than 10% were excluded.

### Subclustering workflow

Certain cell type clusters were subclustered to further inspect their transcriptional heterogeneity. For subclustering, only the clusters of the cell type of interest were retained and the SCT and integrated assays were cleared. Afterwards, the workflow starting from the SCT normalization was repeated. Biological subcluster annotations were based on annotation definitions developed by previous scRNA-Seq and snRNA-Seq datasets that inspected similar cell types (9–12).

### Differential expression and gene set enrichment analysis

Genes differentially expressed in Sarcoidosis relative to control in cell types of interest were calculated by running the FindMarkers() function using the Wilcoxon Rank Sum test. Only genes with an adjusted *p*-value less than 0.05 were retained. Gene set enrichment analysis was conducted by obtaining gene sets associated with each differentially expressed gene via Metascape (15). Gene sets of interest were filtered by keeping gene sets with terms of interest using the grepl() text search command. Afterwards, gene sets of interest were retained by manually ensuring that they are of interest. As gene sets tend to poorly represent the direction of effect genes have on their respective gene sets, the sign of the log_2_[Average Fold Change] for gene set repressors was inverted. Since custom gene sets were developed for this analysis, a custom gene enrichment score had to be computed for the generated gene sets. This gene enrichment score (GES) was calculated by normalizing log_2_[Average Fold Change] of genes in a gene set to the maximum absolute log_2_[Average Fold Change] and summing all the adjusted log_2_[Average Fold Change] such that a positive score would indicate that a gene set was upregulated while a negative score would indicate otherwise.

$$\mathrm{GES}=\;\overset{i\;=\;G}{\underset{i\;=\;1}{\sum }}\frac{\mathrm{lo}g_{2}{\left(\mathrm{avg}\;\mathrm{Fold}\;\mathrm{Change}\right)}_{i}}{\mathrm{MAX}(\left|\mathrm{lo}g_{2}(\mathrm{avg}\;\mathrm{Fold}\;\mathrm{Change})\right|)};\;\;G:\;\mathrm{number}\;\mathrm{of}\;\mathrm{genes}\;\mathrm{in}\;a\;\mathrm{gene}\;\mathrm{set}$$

### Cell communication pathway analysis

The CellChat (16) package was used to run cell-cell communication analysis among subclustered macrophage populations, subclustered endothelial cell populations, and fibroblast populations. The normalized RNA assay was used for the ligand-receptor expression analysis with the labels set to the assigned cell type annotations. Significant pathways were defined as pathways with a communication probability higher than 0.2.

### Transcription factor analysis

Differentially expressed transcription factors (TFs) were determined by separately running the FindMarkers() using the Wilcoxon Rank Sum test for the healthy and diseased samples. Afterwards, a human TF list, downloaded via the SCENIC package (17), was used to only retain TF genes that have an adjusted *p*-value less than 0.05. Subsequently, only TFs deemed to be differentially expressed in Sarcoidosis but not in control conditions were retained.

### Software

All computational work was run via R v4.0.4 with the following packages additionally loaded: scales v1.1.1, lattice v0.20-41, gridExtra v2.3, forcats v0.5.1, ggrepel v0.9.1, ggsignif v0.6.1, ggplot2 v3.3.3, multtest v2.46.0, Biobase v2.50.0, BiocGenerics v0.36.1, BiocManager, v1.30.12, patchwork v1.1.1, SeuratObject v4.0.0, Seurat v4.0.1, tidyr v1.1.3, dplyr v1.0.5, CellChat v1.5.0, igraph v1.3.4. Two-sample student *T* tests were run to execute all additional statistical testing at a 95% level of confidence.

## Results

### Compositional increase of immune and stromal cells in sarcoidosis in the heart and BAL at the cellular level

**Figure 1:**
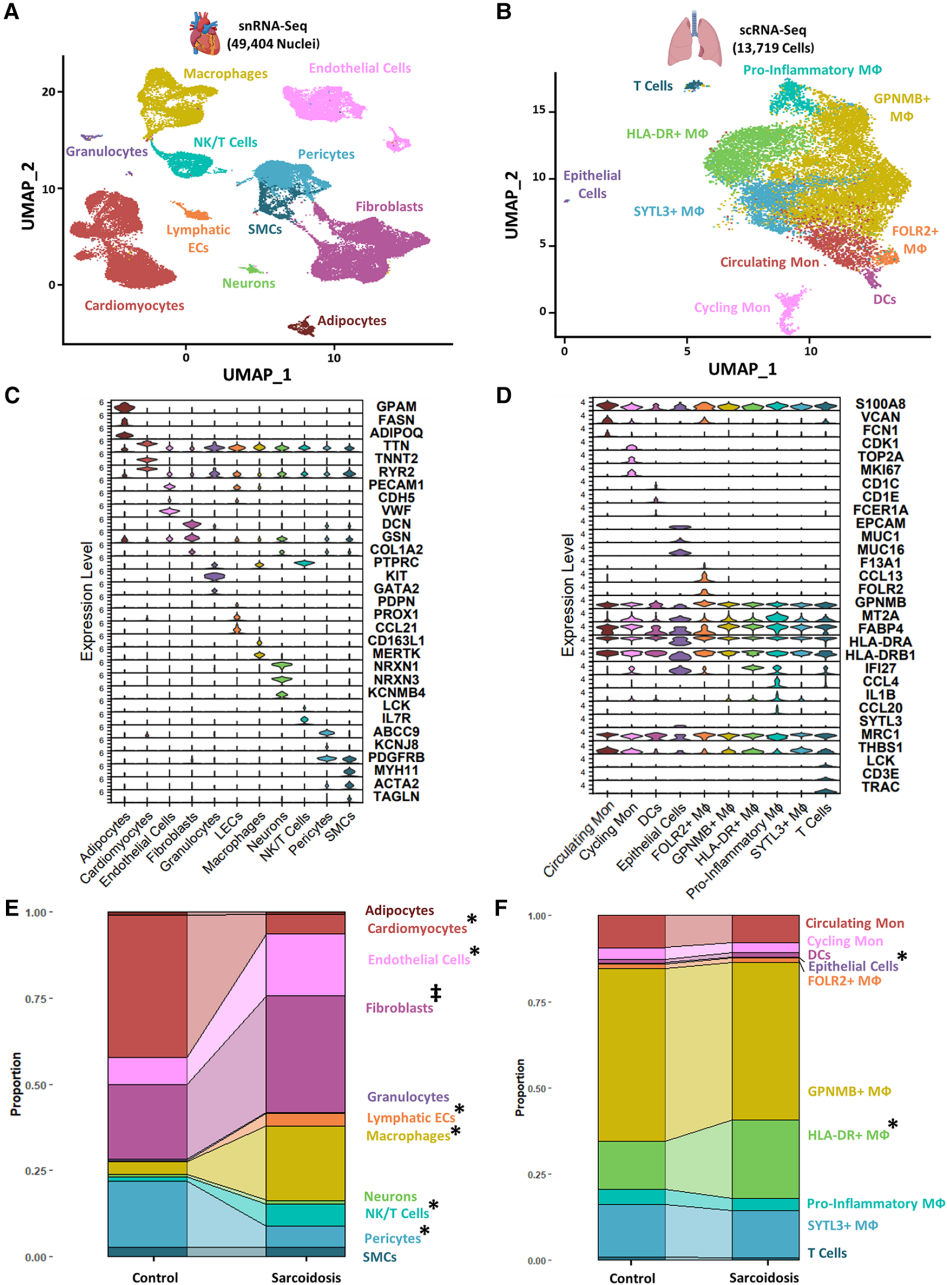
(**A**) UMAP clustering of integrated control and cardiac sarcoidosis (CS) snRNA-Seq datasets by cell type. The following biological cell populations were identified: adipocytes, cardiomyocytes, endothelial cells, fibroblasts, granulocytes, lymphatic ECs, macrophages (MФ), neurons, NK/T cells, pericytes and smooth muscle cells (SMCs). (**B**) UMAP clustering of integrated control and pulmonary sarcoidosis (PS) scRNA-Seq datasets by cell type. The following biological cell populations were identified: circulating monocytes (Mon), cycling Mon, epithelial cells, FOLR2+ MФ, GPNMB+ MФ, HLA-DR+ MФ, pro-inflammatory MФ, SYTL3+ MФ and T cells. (**C**) Violin plot showing the expression of canonical cardiac markers in each identified biological cluster. (**D**) Violin plot showing the expression of canonical BAL markers in each identified biological cluster. (**E**) Proportion analysis of each identified cardiac biological cluster across disease status. (**F**) Proportion analysis of each identified cardiac biological cluster across disease status. Statistically significant changes with a *p*-value <0.05 are indicated by a (*) while statistically trending changes with a *p*-value <0.1 are indicated by (‡).

To examine similarities and differences in cardiac and lung sarcoidosis, we integrated publicly available CS and control cardiac snRNA-Seq datasets (Supplementary Figure S1), which yielded a final Seurat object constituted of 25,224 healthy control cardiac nuclei and 24,180 sarcoidotic cardiac nuclei (Supplementary Figure S2A). We also integrated publicly available PS and control BAL scRNA-Seq datasets (Supplementary Figure S1), which yielded a final Seurat object constituted of 7,423 control BAL cells and 6,296 sarcoidotic BAL cells (Supplementary Figure S2B). We observed immune cells, such as macrophages and T cells in both datasets (Figures 1A,B). Moreover, we identified cardiomyocytes and smooth muscle cells (SMCs) as well as stromal cells, such as endothelial cells, fibroblasts, lymphatic endothelial cells (LECs) and pericytes in the cardiac dataset that were not present in the BAL dataset. In order to confirm that the cell type designations utilized in our analysis represented transcriptionally and biologically distinct clusters, we ran unsupervised differential expression testing (Figures 1C,D, Supplementary Figure S2C, and Supplementary Table S5). Furthermore, to assess the proportional changes of different cell types in the cardiac and BAL Seurat objects, cells in each dataset were separated by disease status and the proportion of each cell type was compared across disease (Figures 1E,F). We found that, relative to control, there was a statistically significant increase in the proportion of macrophages, NK/T cells and endothelial cells in CS. We also noted a statistically significant increase in HLA-DR+ macrophages and epithelial cells in PS relative to control. Furthermore, we observed a statistically significant increase in the proportion of LECs as well as a statistically significant decrease in the proportion of cardiomyocytes and pericytes in CS relative to control (Figures 1E,F). While the *p*-value for the increase in the proportion of fibroblasts relative to control in the cardiac dataset was not statistically significant at a confidence level of 95%, it was less than 0.1 which suggests that there might be a trending increase in that cell type population in CS. Since the total number of cells for each dataset is approximately equal, the previous observations were applicable to cell type numbers of each dataset as well. Due to the multipotent nature of pericytes, we ran pseudotime trajectory analysis on pericytes, SMCs and fibroblasts. We noted that very few pericytes were contracted along the transitional path towards SMCs while most of the pericytes were diminished in sarcoidosis along the early portion of the transitional path towards fibroblasts in CS (Supplementary Figure S2D). Conversely, the proportion of the cells along the later portion of that path were shown to be expanded. Therefore, both sarcoidotic cardiac and BAL samples show a compositional shift towards increased immune cells, such as macrophages and T cells, as well as stromal cells, such as epithelial cells, endothelial cells and fibroblasts.

### Sarcoidotic cardiac and BAL T cells exhibit immune attenuation and dysfunction

**Figure 2:**
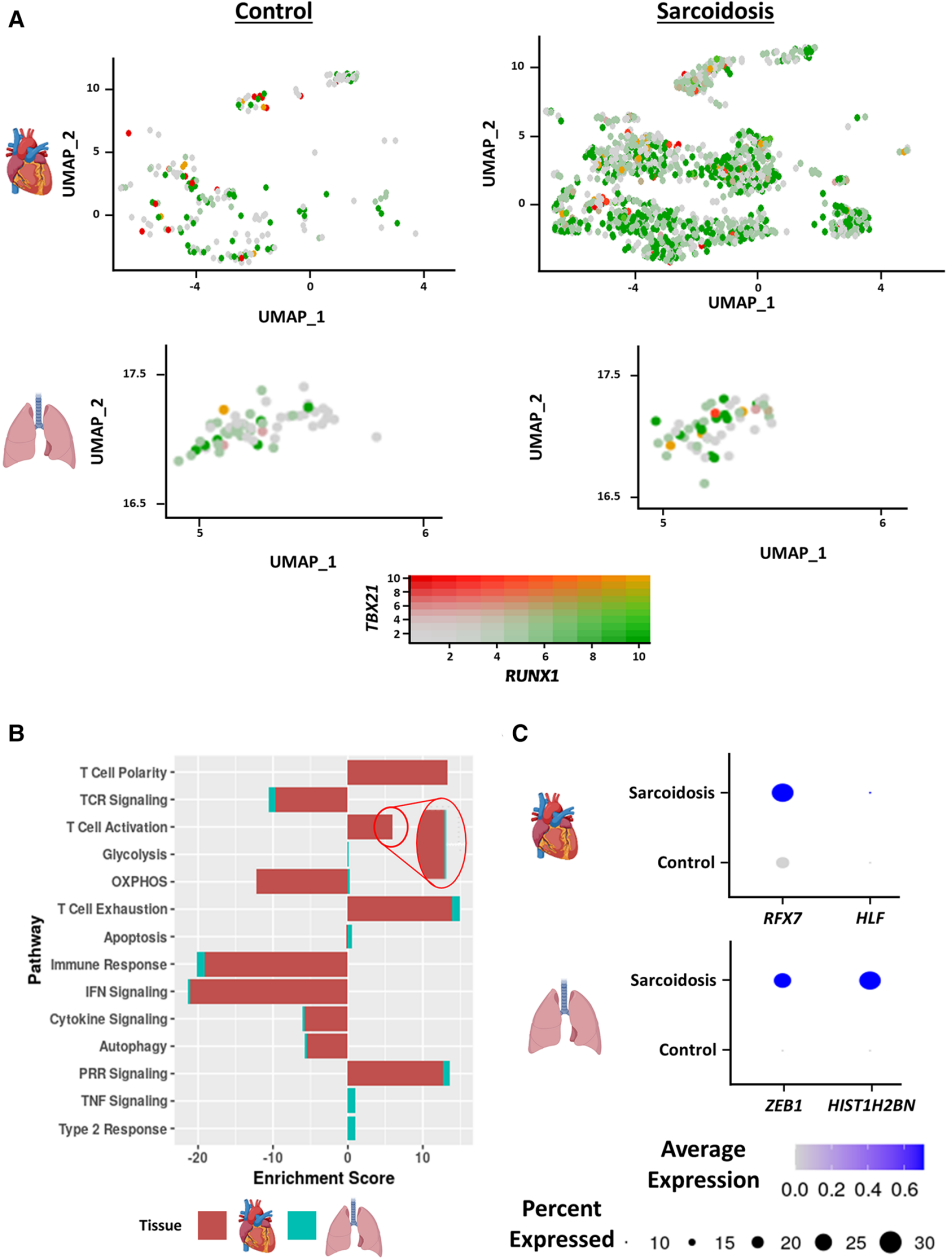
(**A**) Feature plot showing Th17.1 cells co-expressing *TBX21* and *RUNX1* in the cardiac (top) and BAL (bottom) datasets stratified by disease status (control shown on the left, sarcoidosis shown on the right). (**B**) Supervised gene-set enrichment results in cardiac and BAL T cells showing pathways shown to be significantly enriched in sarcoidosis relative to control. Positive enrichment scores indicate pathways shown to be significantly upregulated while negative enrichment scores indicate otherwise. (**C**) Dot plot showing the expression of sarcoidosis-specific transcription factors (TFs) in the cardiac and BAL datasets in control and sarcoidosis T cells. Gene expression was scaled from 0 to 0.6.

Interferon (IFN) γ-producing Th17 cells (Th17.1) are proposed to play a pathogenic role in PS (18). This subset has been functionally defined as CD4^+^ T cells that produce both the Th17 cytokine, IL-17A, as well as the Th1 cytokine, IFNγ (19). To assess the contribution of this subtype in CS as well, we examined the proportion and number of Th17.1 cells, defined as cells/nuclei co-expressing *TBX21* and *RUNX1* in the heart and BAL. This definition has been previously verified as a valid transcriptional definition of Th17.1 (20). Qualitatively, we detected significantly more Th17.1 nuclei in sarcoidosis relative to controls in the heart (Figure 2A). While we saw more sarcoidotic Th17.1 cells in the BAL relative to control, there was not enough Th17.1-containing control donors to determine significance. The reason was that one control donor that yielded the only control BAL Th17.1 cell was determined to be an outlier by running Grubb's test for outliers and was accordingly removed from analysis. We also observed a trending increase in the proportion of Th17.1 cells in sarcoidotic cardiac T cells compared to control cardiac T cells (Supplementary Figure S3A). Both sarcoidotic cardiac and BAL Th17.1 constituted 6%–8% of all sarcoidotic cardiac and BAL T cells (Supplementary Figure S3A). Since none of the remaining control BAL T cells passed the Th17.1 transcriptional definition, we did not run any further comparison with the BAL dataset. Consequently, we ran differential expression testing between all cardiac Th17.1 cells and the other cardiac T cells (Supplementary Table S6). We discerned that only 2 genes were significantly upregulated in Th17.1 relative to all other cardiac T cells: *TBX21*, which encodes T-bet, and *AOAH*, a Th1 response transcription factor (Supplementary Figure S3B). We verified that these two genes were strong Th17.1 markers via ROC analysis (Supplementary Figure S3C and Supplementary Table S6). Hence, we show that both cardiac and BAL sarcoidotic T cells upregulated their composition of Th17.1 cells that are transcriptionally programmed to elicit both a Th1 and Th17 phenotype.

To determine how the transcriptional phenotype of sarcoidotic T cells differs in the heart and BAL, supervised gene-set enrichment was conducted (Supplementary Table S7). We noted that, relative to control, sarcoidotic cardiac and BAL T cells downregulated TCR signaling, immune response, IFN signaling and cytokine signaling transcriptional pathways (Figure 2B). In addition, relative to control, sarcoidotic cardiac and BAL T cells exhibited upregulation of immune dysfunction pathways, such as PRR signaling, autophagy attenuation, T cell activation and exhaustion. While apoptosis and oxidative phosphorylation pathways were upregulated in sarcoidotic BAL T cells, these processes were downregulated in sarcoidotic cardiac T cells (Figure 2B). Other transcriptional distinctions included that, relative to control, only BAL T cells showed upregulation of TNF signaling and type 2 response pathways while only cardiac T cells showed upregulation of the T cell structural polarity pathway (Figure 2B).

To inspect whether there was a shared transcriptional programming pathway for T cells in the heart and BAL, transcription factor (TF) analysis was conducted (Supplementary Table S8). We found that, unlike their control counterparts, both cardiac and BAL sarcoidosis T cells showed enrichment of memory T cell TF genes (*RFX7* and *ZEB1*), survival and proliferation TF genes (*HLF* and *HIST1H2BN*) (Figure 2C) and type 3 response TF genes (*ARNTL* and *ADARB1*) (Supplementary Figure S3D). Meanwhile, only sarcoidotic BAL T cells showed enrichment of *ZNF683*, a tissue residency TF, while only sarcoidotic cardiac T cells showed enrichment of *NR3C1*, a TF that has been associated with dysfunctional terminal T cell activation (Supplementary Figure S3D). Therefore, we demonstrate that both sarcoidotic cardiac and BAL T cells are transcriptionally programmed towards immune dysfunction processes, such as autophagy attenuation, exhaustion and Th17.1 response, as well as the attenuation of immune processes, such as IFN, cytokine and TCR signaling. However, while only sarcoidotic BAL T cells upregulated apoptosis and type 2 pathways, such as oxidative phosphorylation and TNF signaling, only sarcoidotic cardiac T cells exhibited activation priming profiles, indicated by downregulated apoptosis and oxidative phosphorylation as well as upregulated structural T cell polarity.

### Sarcoidotic cardiac and BAL MΦ exhibit attenuated alternative activation

**Figure 3:**
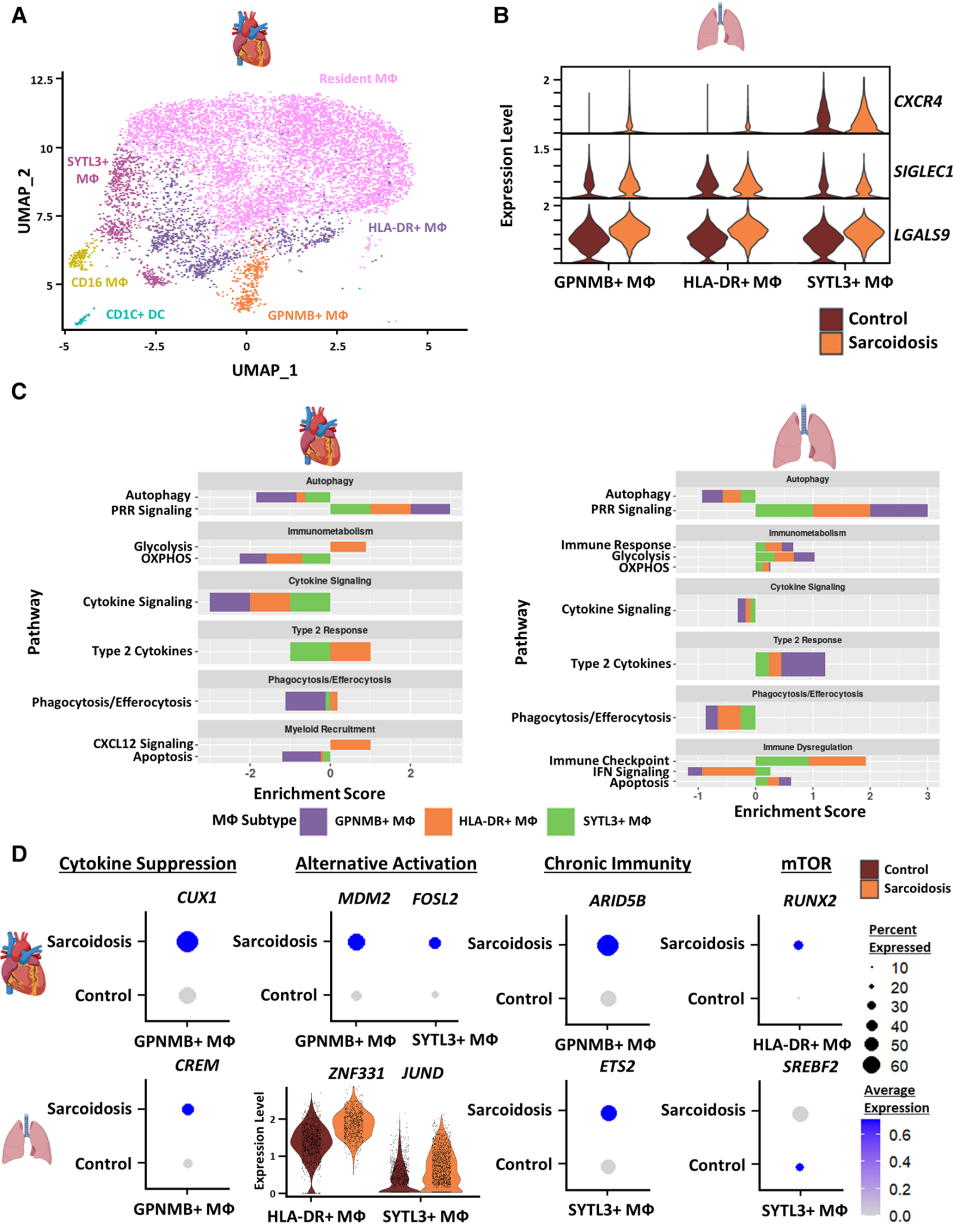
(**A**) UMAP clustering of subclustered cardiac myeloid subpopulations by cell type. The following myeloid subpopulations were identified: Resident MФ, GPNMB+ MФ, HLA-DR+ MФ, CD16 MФ, SYTL3+ MФ and CD1C dendritic cells (DCs). (**B**) Violin plot showing gene expression of cell-cell communication ligands of interest differentially upregulated in sarcoidotic BAL MФ subpopulations stratified by cell type and disease status. (**C**) Supervised gene-set enrichment results in cardiac and BAL MФ subpopulations showing pathways shown to be significantly enriched in sarcoidosis relative to control. Positive enrichment scores indicate pathways shown to be significantly upregulated while negative enrichment scores indicate otherwise. (**D**) Dot plots showing the expression of sarcoidosis-specific transcription factors (TFs) in the cardiac and BAL datasets in control and sarcoidosis MФ subpopulations. Average gene expression was scaled from 0 to 0.6. The expression of *ZNF331* and *JUND* was visualized using a violin plot stratified by cell type and disease status for aesthetic purposes. The classification group of each TF is shown colored above its respective gene expression plot.

To inspect similarities and differences between macrophages (MΦ) in CS and PS, we subclustered cardiac MΦ into 6 subgroups: CD16 MΦ, Glycoprotein Nmb (GPNMB)+ MΦ, HLA-DR+ MΦ, resident MΦ, Synaptotagmin Like 3 (SYTL3)+ MΦ as well as CD1C+ dendritic cells (DCs) (Figure 3A). These annotations have been previously validated in cardiac MΦ by a previous transcriptomic study of CS (9). We further verified these MΦ subgroup annotations using canonical cell type markers as well as unsupervised differential expression testing (Supplementary Figures S4A,B, and Supplementary Table S9). These MΦ subpopulations were identifiable in the BAL dataset without further need for subclustering (Figure 1A). To inspect interactions between these subsets, we ran cell-cell communication analysis in cardiac and BAL MΦ (Supplementary Figure S4C, Supplementary Table S10). We observed that the expression of *CXCR4* [receptor for Chemokine CXC Motif Ligand 12 (CXCL12)], *SIGLEC1* (IFN-related factor), and *LGALS9* (immune checkpoint ligand) were upregulated in sarcoidotic BAL MΦ relative to control BAL MΦ (Figure 3B).

To further assess whether certain transcriptional pathways are conserved in sarcoidotic MΦ in the heart and BAL, we conducted supervised gene-set enrichment (Supplementary Table S11). We found that, relative to control, both cardiac and BAL sarcoidosis MΦ exhibited upregulated alternative activation and glycolysis pathways as well as downregulated cytokine signaling, phagocytosis/efferocytosis and autophagy pathways (Figure 3C). Gene-set enrichment showed that certain pathways were, however, tissue-specific in sarcoidotic MΦ. Relative to control, sarcoidotic cardiac MΦ downregulated apoptosis, but apoptosis was upregulated in sarcoidotic BAL MΦ (Figure 3C). In addition, relative to control, we noted that CXCL12 signaling was upregulated only in sarcoidotic cardiac MΦ while immune checkpoint signaling was upregulated only in sarcoidotic BAL MΦ. Additionally, only sarcoidotic BAL MΦ displayed downregulation of IFN signaling relative to control (Figure 3C, right).

To assess whether the transcriptional pathway results could be corroborated at the level of TF expression, we conducted TF analysis of sarcoidosis-specific TFs (Supplementary Table S12). We observed that, unlike their control counterparts, both sarcoidotic BAL and cardiac GPNMB+ MΦ enriched cytokine suppression TFs (*CREM* and *CUX1* as well as *NR1H3*) and alternative activation TFs (*JUND* and *ZNF331* in BAL MΦ as well as *MDM2* and *FOSL2* in cardiac MΦ) (Figure 3D and Supplementary Figure S4D). Other TFs enriched only in sarcoidotic BAL and cardiac MΦ included chronic immunity TFs (*ETS2* by BAL SYTL3+ MΦ and *ARID5B* by cardiac GPNMB+ MΦ) as well as mTOR TFs (*SREBF2* by BAL SYTL3+ MΦ and *RUNX2* by cardiac HLA-DR+ MΦ) (Figures 3D). Only sarcoidotic cardiac MΦ, however, revealed enrichment of *VDR*, a macrophage recruitment TF (Supplementary Figure S4E). Therefore, we show that, relative to control, both cardiac and BAL sarcoidotic MΦ exhibited attenuated alternative activation transcriptional phenotypes, as determined by the suppression of cytokine signaling, autophagy and phagocytosis/efferocytosis pathways, as well as by the simultaneous upregulation of glycolysis and type 2 response pathways. However, relative to control, only cardiac sarcoidotic MΦ presented with upregulation of the chemotaxis pathway and downregulation of the apoptosis pathway. Conversely, relative to control, only BAL MΦ displayed upregulation of immune checkpoint and apoptosis pathways as well as downregulation of the IFN signaling pathway.

### Fibroblasts exhibit upregulated fibrotic, pro-inflammatory, and dysfunctional activation profiles in CS

**Figure 4:**
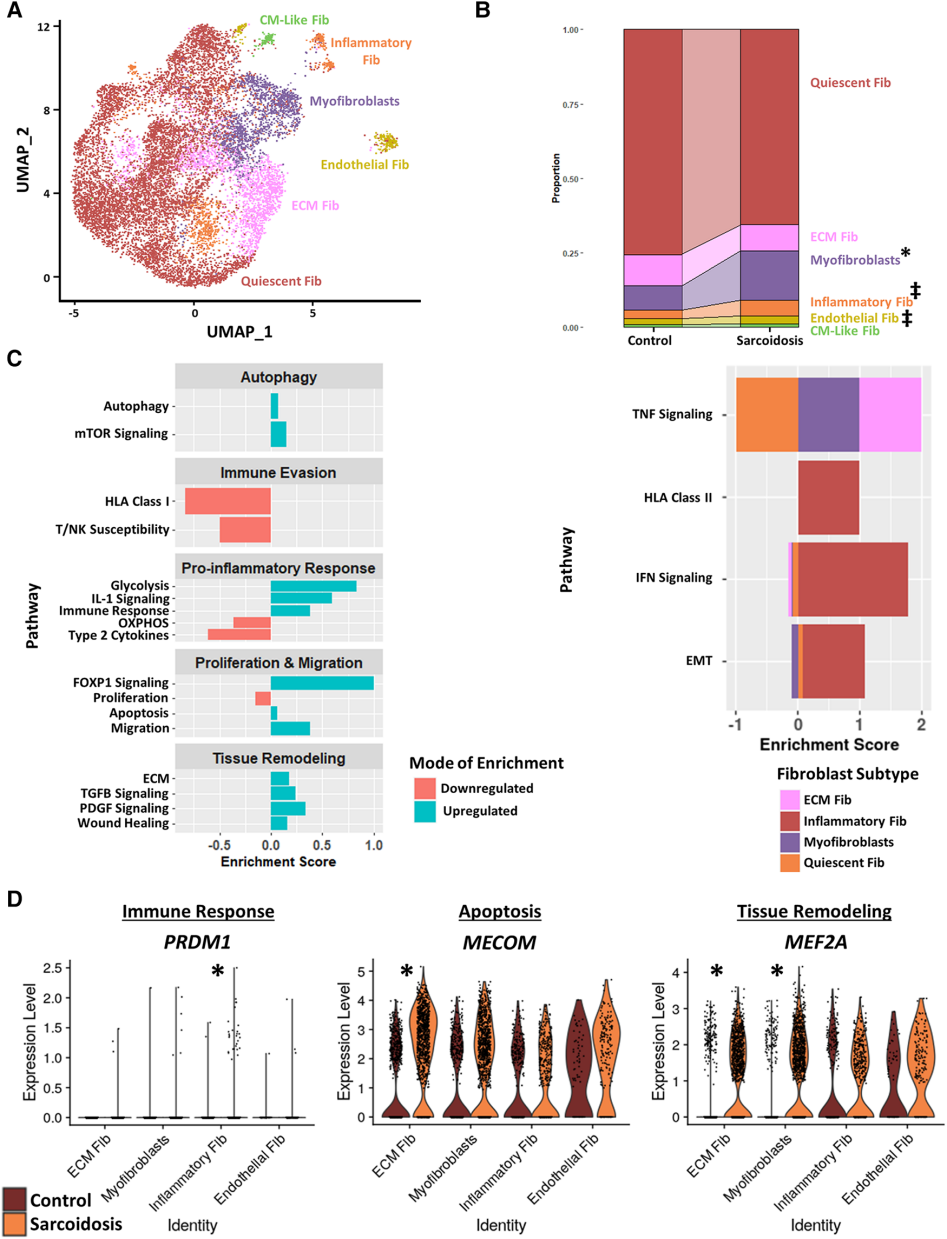
(**A**) UMAP clustering of subclustered cardiac fibroblast subpopulations by cell type. The following fibroblast subpopulations were identified: quiescent fibroblasts (Fib), ECM Fib, myofibroblasts, inflammatory Fib, endothelial Fib and CM-like Fib. (**B**) Proportion analysis of each identified cardiac fibroblast cluster across disease status. Statistically significant changes with a *p*-value <0.05 are indicated by a (*) while statistically trending changes with a *p*-value <0.1 are indicated by (‡). (**C**) Supervised gene-set enrichment results in cardiac fibroblast subpopulations showing pathways shown to be significantly enriched in sarcoidosis relative to control. Pathways shown to be significantly enriched in all fibroblast clusters are shown on the left while subpopulation-specific patterns are shown on the right. Positive enrichment scores indicate pathways shown to be significantly upregulated while negative enrichment scores indicate otherwise. (**D**) Violin plots stratified by cell type and disease status showing the expression of sarcoidosis-specific transcription factors (TFs) in the cardiac datasets in control and sarcoidosis fibroblast subpopulations. The classification group of each TF is shown colored above its respective gene expression plot.

To further investigate cardiac fibroblasts in CS, we subclustered cardiac fibroblasts into 6 biologically relevant and transcriptionally distinct fibroblast clusters: quiescent fibroblasts, ECM fibroblasts which were defined as fibroblasts highly expressing extracellular matrix (ECM)-related genes, myofibroblasts which were defined as fibroblasts highly expressing activation markers, inflammatory fibroblasts which were defined as fibroblasts highly expressing inflammatory markers, endothelial fibroblasts which were defined as fibroblasts highly expressing endothelial cell markers, and cardiomyocyte (CM)-like fibroblasts which were defined as fibroblasts highly expressing CM markers (Figure 4A, Supplementary Figures S5A,B, and Supplementary Table S13). We ran cell proportion analysis and showed that there was a statistically significant increase in the proportion of myofibroblasts (Figure 4B). We also found a trending increase in the proportion of inflammatory and endothelial fibroblasts.

Subsequently, we conducted supervised gene-set enrichment to assess the transcriptional phenotypes of sarcoidotic cardiac fibroblasts (Supplementary Table S14). We observed that, relative to control, all sarcoidotic cardiac fibroblasts exhibited an increased pro-inflammatory transcriptional phenotype, as indicated by upregulated glycolysis, immune response and IL-1 signaling as well as downregulated HLA class I, susceptibility to T/NK mediated cytotoxicity, type 2 cytokines and oxidative phosphorylation (Figure 4C, left). Other transcriptional phenotypes differentially upregulated by sarcoidotic cardiac fibroblasts included tissue remodeling processes, such as ECM, Transforming Growth Factor β (TGFβ) and Platelet-Derived Growth Factor (PDGF) signaling and wound healing, as well as dysfunctional activation processes, such as autophagy, mammalian target of rapamycin (mTOR) signaling, Forkhead Box Protein P1 (FOXP1) signaling, apoptosis, migration and FOXP1 signaling. Only sarcoidotic inflammatory fibroblasts, however, upregulated HLA class II as well as IFN signaling pathways, relative to control (Figure 4C, right). Similarly, only ECM fibroblasts and myofibroblasts upregulated the TNF signaling pathway, relative to control. While only quiescent and inflammatory fibroblasts showed upregulation of the epithelial-mesenchymal transition pathway, relative to control (Figure 4C, right).

To assess how these enrichment analysis results corroborated with TF trends, we ran TF analysis (Supplementary Table S15). We observed that, unlike their control counterparts, only sarcoidotic inflammatory fibroblasts showed enrichment for immune response TFs, such as *PRDM1*, only sarcoidotic ECM fibroblasts showed enrichment of apoptosis TF, such as *MECOM*, and only sarcoidotic myofibroblasts and ECM fibroblasts showed enrichment of tissue remodeling TFs, such as *MEF2A* (Figure 4D). Unlike myofibroblasts and ECM fibroblasts which enriched fibroblast activation TFs, such as *GLIS1* and *TRPS1*, only in sarcoidosis, endothelial fibroblasts showed enrichment of *FLI1*, a fibroblast activation suppressor TF, and *GATA2*, an endothelial cell TF, strictly in sarcoidosis (Supplementary Figure S5C). Therefore, we show via gene-set enrichment and TF analysis that CS promotes transcriptional profiles characterized by upregulated tissue remodeling processes, such as ECM, TGFβ and PDGF signaling and wound healing, upregulated immunomodulatory processes, such as glycolysis, immune evasion and type 1 response, and dysfunctional activation, characterized by upregulated autophagy, apoptosis as well as migration.

### Endothelial cells exhibit more immune modulation, angiogenesis and mTOR signaling in CS

**Figure 5:**
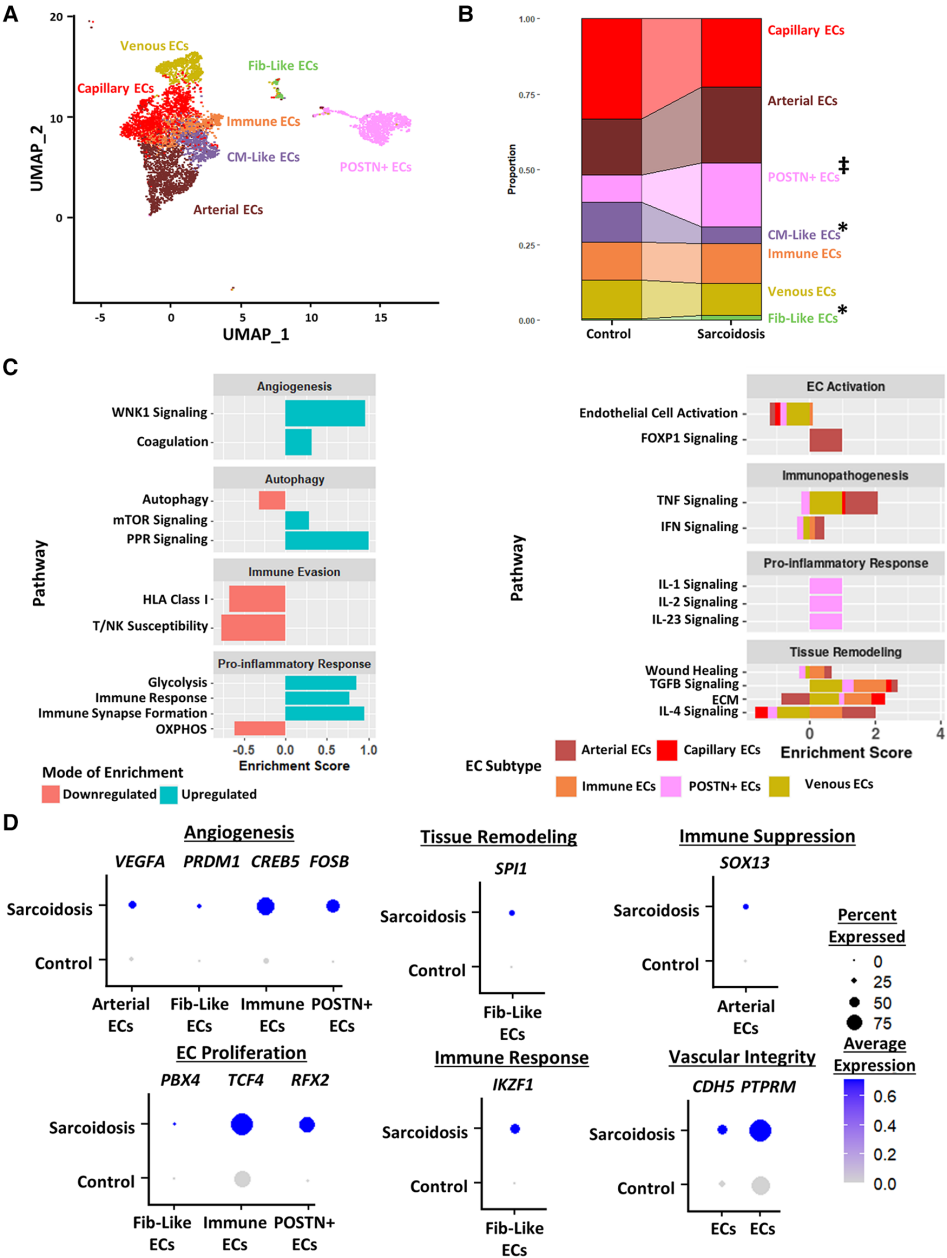
(**A**) UMAP clustering of subclustered endothelial cell subpopulations by cell type. The following fibroblast subpopulations were identified: capillary endothelial cells (ECs), arterial ECs, POSTN+ CM-like ECs, immune ECs, venous ECs and Fib-like ECs. (**B**) Proportion analysis of each identified cardiac endothelial cell cluster across disease status. Statistically significant changes with a *p*-value <0.05 are indicated by a (*) while statistically trending changes with a *p*-value <0.1 are indicated by (‡). (**C**) Supervised gene-set enrichment results in cardiac endothelial subpopulations showing pathways shown to be significantly enriched in sarcoidosis relative to control. Pathways shown to be significantly enriched in all endothelial cell clusters are shown on the left while subpopulation-specific patterns are shown on the right. Positive enrichment scores indicate pathways shown to be significantly upregulated while negative enrichment scores indicate otherwise. (**D**) Dot plots stratified showing the expression of sarcoidosis-specific transcription factors (TFs) in the cardiac datasets in control and sarcoidosis endothelial cell subpopulations. Average gene expression was scaled from 0 to 0.6. The classification group of each TF is shown colored above its respective gene expression plot.

To further study endothelial cells in CS, we subclustered cardiac endothelial cells into 7 transcriptionally distinct subtype clusters: capillary endothelial cells which highly express capillary markers, arterial endothelial cells which highly express arterial markers, Periostin (POSTN)+ endothelial cells, cardiomyocyte (CM)-like endothelial cells which highly express CM markers, immune endothelial cells which highly express immunomodulatory markers, venous endothelial cells which highly express venous markers, and fibroblast-like endothelial cells which highly express tissue remodeling markers (Figure 5A, Supplementary Figures S6A,B, Supplementary Table S16). While epithelial cells were identified in the BAL dataset, they were not detected in CS. We noted via cell proportion analysis that there was a statistically significant increase in fibroblast-like endothelial cells and a statistically significant decrease in CM-Like endothelial cells (Figure 5B). We also found a trending increase in the proportion of POSTN+ endothelial cells.

Differential expression and supervised gene-set enrichment testing was conducted in each sarcoidotic endothelial cell type relative to its control counterpart (Supplementary Table S17). As there was not enough control fibroblast-like endothelial cells to compare sarcoidotic Fib-like endothelial cells against, fibroblast-like endothelial cells were removed from enrichment analysis. In addition, CM-like endothelial cells were discarded from further analysis as there was an insufficient number of sarcoidotic CM-like endothelial cells. Regardless of subtype, all analyzed sarcoidotic endothelial cells showed increased immune transcriptional activation relative to control, as indicated by upregulation of glycolysis, immune response and immune synapse formation as well as by downregulation of HLA class I, susceptibility to T/NK-mediated cytotoxicity and oxidative phosphorylation (Figure 5C, left). Other transcriptional phenotypes included upregulation of angiogenesis pathways, such as coagulation and With No Lysine/K Lysine Deficient Protein Kinase 1 (WNK1) signaling, and downregulation of mTOR-dependent autophagy. While sarcoidotic arterial endothelial cells showed upregulation of TNF and IFN signaling pathways, they exhibited downregulation of endothelial cell activation pathways, as indicated by upregulated FOXP1 signaling (Figure 5C, right). Additionally, most endothelial cells, except for immune and POSTN+ endothelial cells, displayed upregulation of tissue remodeling pathways, such as TGFβ signaling and ECM. While sarcoidotic POSTN+ endothelial cells did not show clear upregulation of tissue remodeling pathways, they were the only subtype to upregulate pro-inflammatory pathways, such as IL-1, IL-2 and IL-23 signaling (Figure 5C, right).

To further assess transcriptional programs utilized by sarcoidotic endothelial cells, TF analysis was conducted (Supplementary Table S18). As venous and capillary endothelial cells mostly represent anatomical niches that are unlikely to be functionally unique in CS (Figure 5C), these subtypes were discarded from subsequent TF analysis. We found that, unlike their control counterparts, all analyzed sarcoidotic endothelial cells highly expressed unique angiogenic TFs, such as *VEGFA*, *PRDM1*, *CREB5* and *FOSB* (Figure 5D). We also found that, unlike their control counterparts, all analyzed sarcoidotic endothelial cells (except for arterial endothelial cells) were enriched for unique proliferation TFs, such as *PBX4*, *TCF4* and *RFX2*. Conversely, unlike their control counterparts, only sarcoidotic fibroblast-like endothelial cells displayed enrichment of *SPI1*, a tissue remodeling TF, and *IKZF1*, an immunomodulatory TF (Figure 5D). Meanwhile only sarcoidotic arterial endothelial cells were enriched for *SOX13*, an immunosuppressive TF. Additionally, all sarcoidotic endothelial cells showed upregulation of *CDH5* and its receptor *PTPRM* relative to control, as was indicated by cell-cell communication analysis (Figure 5D, Supplementary Figures S6C,D). Therefore, we demonstrate using gene-set enrichment and TF analysis that sarcoidotic endothelial cells transcriptionally upregulate mTOR signaling, immunomodulatory processes, such as glycolysis and immune response, as well as angiogenesis, characterized by CDH5-PTPRM communication. However, sarcoidotic endothelial cells exhibit heterogeneity in promoting tissue remodeling and cytokine signaling, as we showed that while the TNF signaling pathway was upregulated by arterial and immune endothelial cells, the IFN signaling pathway was uniquely upregulated by immune endothelial cells.

### CS promotes pro-inflammatory, cardiotonic and proliferative profiles in cardiomyocytes

**Figure 6:**
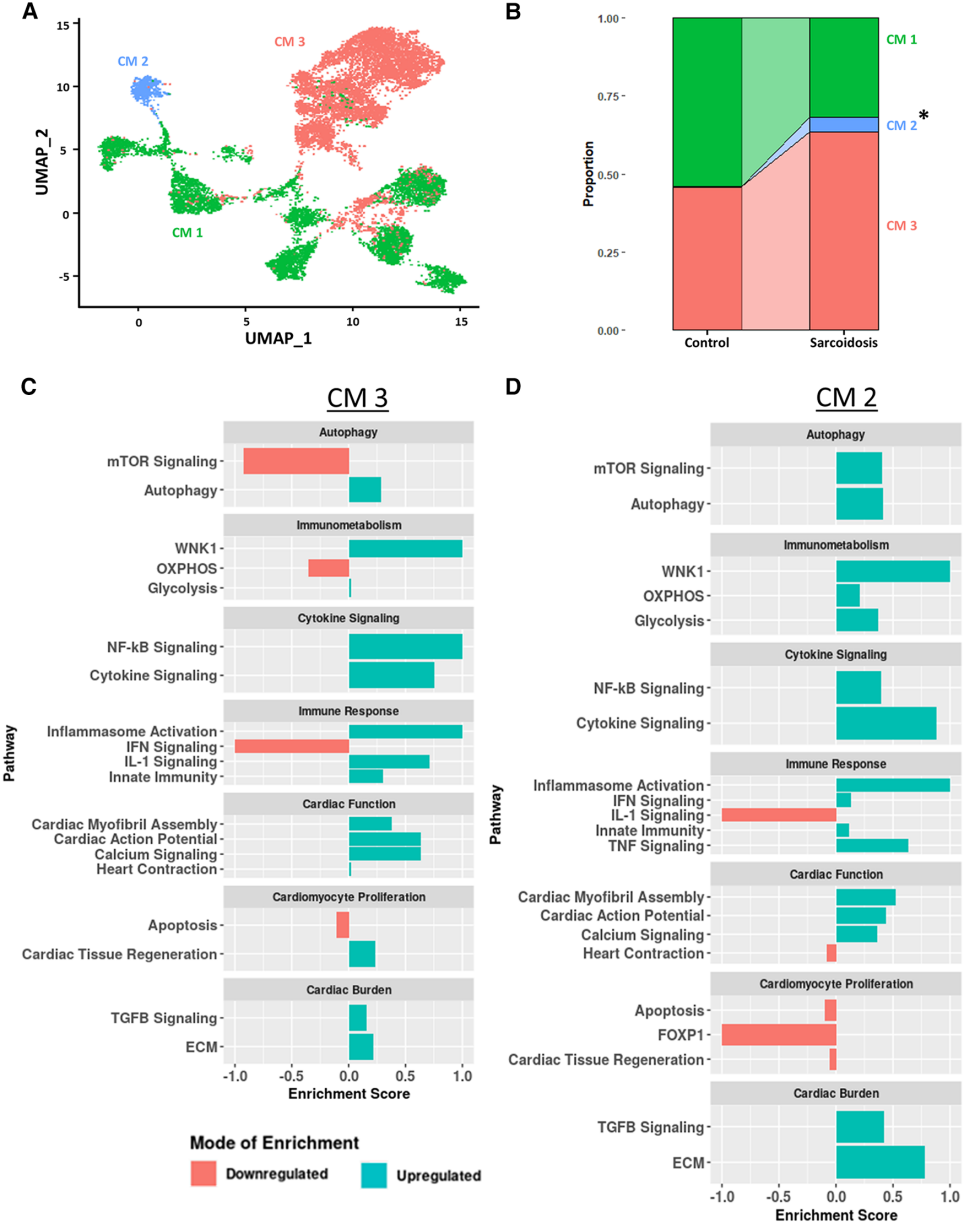
(**A**) UMAP clustering of subclustered cardiomyocyte subpopulations by cell type. Three distinct cardiomyocyte subclusters were identified termed: CM 1, CM 2 and CM 3. (**B**) Proportion analysis of each identified cardiomyocyte subcluster across disease status. Statistically significant changes with a *p*-value <0.05 are indicated by a (*). (**C**) Supervised gene-set enrichment results in cluster CM 3 showing pathways shown to be significantly enriched in CM 3 relative to CM 1 and CM 2. Positive enrichment scores indicate pathways shown to be significantly upregulated while negative enrichment scores indicate otherwise. (**D**) Supervised gene-set enrichment results in cluster CM 2 showing pathways shown to be significantly enriched in CM 2 relative to CM 1 and CM 3. Positive enrichment scores indicate pathways shown to be significantly upregulated while negative enrichment scores indicate otherwise.

Due to the cardiac symptomology of CS, we next investigated the transcriptomic changes in sarcoidotic cardiomyocytes. Despite integration and harmony-based batch effect correction, most sarcoidotic cardiomyocytes identified initially in the heart (Figure 1A) clustered in a cluster termed “CM 3” while most control cardiomyocytes clustered in a cluster termed “CM 1” (Supplementary Figure S7A). Additionally, there was a third distinct cardiomyocyte cluster termed “CM 2”, constituted mostly of sarcoidotic cardiomyocytes. To further analyze these three clusters, cardiomyocytes were isolated from the heart dataset for more granular integration and batch correction as well as for sub-clustering. Even after re-clustering, the three cardiomyocyte clusters were still spatially segregated by disease status (Figure 6A). Differential expression testing showed that the three clusters were transcriptionally distinct (Supplementary Figure S7B, Supplementary Table S19). Cell proportion analysis revealed that while there was no statistically significant difference in the proportion of cells in the CM 1 and CM 3 clusters across disease, there was a statistically significant increase in the proportion of cells in the CM 2 cluster in CS relative to control (Figure 6B).

To assess the functional heterogeneity of these cardiomyocyte clusters, we conducted supervised gene-set enrichment testing (Supplementary Table S20). We showed that CM 2 and CM 3 showed differential immune response activation, as indicated by upregulated WNK1 signaling, glycolysis, cytokine signaling, Nuclear Factor Kappa-light-chain-enhancer of Activated B (NF-κB) signaling, innate immunity and inflammasome activation (Figures 6C,D). CM 2 and CM 3 also displayed upregulated cardiac burden transcriptional processes, such as ECM and TGFβ signaling, and cardiac function, as indicated by upregulated calcium signaling, cardiac action potential, and cardiac myofibril assembly. In addition, CM 2 and CM 3 showed differential transcriptional upregulation of cardiomyocyte proliferation processes, as shown by downregulated apoptosis. While CM 3 showed upregulated IL-1 signaling, heart contraction and cardiac tissue regeneration, these pathways were downregulated in CM 2. Conversely, while mTOR and IFN signaling were downregulated in CM 3, these pathways were upregulated in CM 2. Furthermore, certain enrichment patterns, such as upregulation of TNF signaling and downregulation of FOXP1 signaling, a critical TF for cardiomyocyte proliferation, were unique to CM 2. These results were further corroborated by the observation that *TNNI3*, encoding for troponin I, was highly downregulated in CM (Supplementary Figure S7C). Therefore, we show that, despite transcriptional heterogeneity, sarcoidotic cardiomyocytes transcriptionally promote immune response processes, such as glycolysis, cytokine signaling, innate immunity and type 1 response, cardiac function processes, such as calcium signaling, heart contraction, cardiac action potential and cardiac myofibril assembly, and stress response processes, such as cardiac burden and cardiac tissue regeneration, as well as proliferation processes, via downregulating apoptosis.

## Discussion

CS presents a significant public health burden, with high mortality risk (3, 4). However, the pathogenesis of this disease is largely understudied. In this transcriptomic study, we dissected the transcriptional complexity of major cardiac resident and immune cell types that might be involved in CS pathogenesis, comparing them to counterpart BAL cell types when possible. For this purpose, we separately integrated healthy and sarcoidosis cardiac snRNA-Seq as well as BAL scRNA-Seq datasets. We observed an expansion in immune (myeloid and lymphoid) as well as stromal populations, such as fibroblasts and endothelial cells, in both PS and CS, relative to control. This finding was in line with what has been shown about granuloma structure in different forms of sarcoidosis, such as renal and pancreatic sarcoidosis (21, 22). Interestingly, CS exhibited a statistically significant decrease in the proportion of pericytes relative to healthy heart control. As pericytes are known to differentiate into myofibroblasts (23), it could be argued that the observed reduction in pericytes might indicate that a portion of cardiac pericytes could be redirected into the fibroblast population in CS. This is corroborated by our findings that the fibroblast population, specifically myofibroblasts, were shown to be significantly expanded in CS relative to control. This hypothesis was further supported by our trajectory analysis of pericytes and fibroblasts that showed that the proportion of cells midway through the pericyte-fibroblast differentiation trajectory were higher in CS relative to control.

While it has been shown that T cells are a major constituent of sarcoidotic granulomas (1), studies investigating the pathogenic role of T cells in CS are lacking. Recently, there has been a paradigm shift from Th1 to Th17.1 (which are IFNγ+ IL-17A+) as the pathogenic T cell population in PS (18). This subset has also been implicated as potential mediators of sarcoidosis-like complications following CAR T cell treatment (24). Here, we show that Th17.1, which we defined based on previous *in vitro* work on IL-17A+ IFNγ+ T cells as *TBX21*+ *RUNX1*+ (20), expand in CS relative to control as well. While this expansion was only trending in PS, that might be due to the myeloid bias of BAL samples. We also showed that, unlike their BAL counterparts, cardiac Th17.1 are best defined as *TBX21*+ *AOAH*+. This was reasonable as *TBX21* and *AOAH* mediate Th1 polarization, a known characteristic of Th17.1. Additionally, we showed via gene-set enrichment and transcription factor analysis that CS and PS exhibit downregulated immune response, cytokine and TCR signaling as well as upregulated chronic exhaustion pathways relative to control. This was well-corroborated by other studies that reported that BAL-derived T cells exhibited low proliferative and cytokine signaling phenotypes in PS (25). While there was a study that reported increased Th1 cytokine signaling in CS (26), it was mostly focused on active disease and cytokine levels assessed via bulk measurements throughout large histological samples. While we observed that only BAL T cells upregulated apoptosis, TNF signaling and type 2 response processes, such as OXPHOS, relative to control, which has been previously reported in the PS literature (25, 27, 28), cardiac T cells were shown to downregulate OXPHOS with insufficient glycolysis compensational upregulation. Metabolic energy is critical for T cell activation and differentiation (29). Such metabolic insufficiency has been linked with quiescent and suppressed T cells (30). This transcriptional metabolic trend is well-corroborated with the fact that we observed downregulated activation profiles in CS T cells relative to their control counterparts. However, more protein and functional T cell studies in CS are needed to properly elucidate this transcriptional phenotype. Nevertheless, we show that both PS and CS T cells exhibit an attenuated activation profile as well as expansion of Th17.1 populations relative to control.

There has been a recent renewed interest in the pathogenic role of macrophages, a core constituent of sarcoidotic granulomas. For instance, a single cell transcriptomic analysis on cardiac macrophages in CS and ischemic cardiomyopathy has recently been reported (9), focusing mostly on the upregulation of mTOR signaling in CS. However, the pathogenic role of macrophages in sarcoidosis still remains unclear. As such, we aimed to elucidate other pathways involved in this pathogenic role. Gene-set enrichment and transcription factor analysis revealed that both CS and PS MΦ exhibit upregulated alternative activation yet attenuated effector function, as indicated by downregulated cytokine signaling and phagocytosis, relative to control. Similar observations have been previously reported in PS. For instance, previous studies have noted that, especially during the chronic stage of PS, BAL MΦ tend to exhibit alternative activation phenotypes (27) and attenuated phagocytic function (31). However, while it is still uncertain if such a phenotype is present in CS, there has been a report that CS granulomas tend to express Folate Receptor-β (FOLR2) which has been recently implicated in alternative activation polarization in cardiac MΦ (32, 33). Additionally, we observed downregulation of OXPHOS without sufficient glycolysis compensational upregulation only in CS MΦ. Such metabolic stress has been shown to drive macrophage alternative and activation (34) and to dysregulate their phagocytic function (35). This could explain our transcriptional observations that imply that CS MΦ might have impaired phagocytic function and be more polarized towards alternative activation. Intriguingly, only BAL MΦ exhibited upregulation of immune checkpoint signaling relative to control. This finding was well-corroborated by recent findings that T cells and NK cells upregulate the expression of immune checkpoint molecules, such as PD-1, CTLA-4 and TIGIT (36). In addition, this is consistent with several case reports that describe patients that developed immunotherapy-induced lung sarcoidosis with upregulated involvement of giant cell macrophages (37, 38). Conversely, only CS MΦ exhibited upregulated CXCL12 signaling but attenuated apoptosis relative to control. This could explain the study reporting high FOLR2 expression in CS MΦ as FOLR2 is also a marker of tissue resident MΦ (32, 33) which are known to exhibit tissue recruitment and self-renewal. Hence, we show that despite tissue-specific differences, both PS and CS MΦ exhibit attenuated alternative activation profiles relative to control.

In addition to inspecting immune cells, we investigated the transcriptomic signatures of stromal cells implicated in CS pathogenesis as well. One such cell subset is that of cardiac fibroblasts. Besides the observed increase in the fibroblast proportion, we noted a significant increase in the frequency of myofibroblasts as well as endothelial and inflammatory fibroblasts relative to control. Gene-set enrichment and transcription factor analysis revealed that all cardiac fibroblast subsets exhibited upregulation of pro-inflammatory and pro-fibrotic phenotypes relative to control. This is corroborated by studies that showed that CS patient cardiac histological samples exhibit high degrees of fibrosis as well as PET imaging studies that report increased fibroblast activation in CS (3, 39). Links between pro-inflammatory phenotypes and fibroblast activation in CS has previously been shown as it relates to monocyte-derived macrophages (40). This might be especially true since we have shown that only inflammatory fibroblasts exhibited upregulation of IFN signaling and HLA Class II signaling, a product of IFN signaling and a sign of fibroblast activation. This might be an important phenotype as IFN signaling has been implicated in the pathogenesis of PS (41) and could be implicated in CS as well. Intriguingly, we observed upregulation of apoptosis and autophagy by all cardiac fibroblast subsets. Autophagy has been shown to be downregulated by immune cells implicated in CS pathogenesis (9), such as cardiac MΦ. This finding implies that autophagy attenuation might be cell specific. Thence, we show that CS cardiac fibroblasts exhibit upregulated pro-fibrotic and pro-inflammatory profiles relative to control.

The second stromal cell population we examined was cardiac endothelial cells. In addition to the observed increase in the endothelial cell proportion, we observed a significant increase in POSTN+ and fibroblast-like endothelial cells as well as a significant decrease in cardiomyocyte-like endothelial cells relative to control. Fibrosis within blood vessel walls has been reported in CS patients, particularly in the aorta and coronary arteries (42, 43). It could be postulated that there could be a link between this histopathological feature of CS and the expansion of fibroblast-like endothelial cells. While angiogenesis has not explicitly been investigated before in CS, dysfunction in cardiac microvasculature, aortic elastic properties and coronary flow reserve have been reported in CS patients (43–45). Moreover, upregulated angiogenesis has been reported in other forms of sarcoidosis, such as neurosarcoidosis and PS (46, 47). Our analysis showed upregulation of angiogenic pathways in both our gene-set enrichment and transcription factor analysis relative to control. This was further supported by the expansion of POSTN+ endothelial cells in CS relative to control which have been shown to be primary mediators of pathological angiogenesis in various disease models (48). We also showed that cardiac endothelial cells upregulate immune response and evasion pathways in CS. Particularly, POSTN+ endothelial cells upregulated angiogenic cytokine signaling, such as IL-23 and IL-1 signaling. This is supported by studies that showed that cardiomyocytes in CS patients upregulate the expression of tissue factor pathway inhibitor (TFPI) which is implicated in IL-1 signaling as well as angiogenesis (49). In addition, there have been several case reports describing CS patients who develop vasculitis towards the end-stage of their disease (50, 51). This suggests that CS might be mediating this endothelial cell inflammation. We also detected an mTOR-dependent downregulation of autophagy in endothelial cells in CS relative to control. While autophagy defects have been long implicated in sarcoidosis pathology, the canonical thinking is in how this pathway is defected in immune cells, such as MΦ. In fact, mTOR signaling defects in cardiac MΦ have already been implicated in CS and PS pathology (9, 52). Here, we show that this defect might not be specific to immune cells. Another non-canonical observation was that all cardiac endothelial cells upregulated tissue remodeling pathways, such as TGFβ and ECM signaling, relative to control. This is certainly not a novel thought as many studies have showed that endothelial cells can have pro-fibrotic functions (53), but this suggests that histological fibrosis reported in CS might be the result of the concerted action of fibroblasts and endothelial cells. Thus, we show that cardiac endothelial cells exhibit upregulated angiogenic and pro-inflammatory phenotypes in CS relative to control.

The last cell type we inspected was cardiomyocytes. We observed that sarcoidotic cardiomyocytes were transcriptionally distinct from control cardiomyocytes. Importantly, sarcoidotic cardiomyocytes exhibited upregulated cardiac function processes, such as heart contraction and cardiac action potential, as well as pro-inflammatory and proliferative phenotypes relative to control. This was corroborated by studies that showed that CS cardiomyocytes upregulate the expression of TFPI which mediates IL-1 signaling (49). While previous reports have shown that CS is characterized by destruction of cardiomyocyte tissue (54), it is possible that the noted upregulation in proliferation and cardiac function profiles might reflect that the remaining viable cardiomyocytes promote such transcriptional pathways as a compensatory mechanism. This might be particularly true as we have shown that the proportion of cardiomyocytes are significantly reduced by as much as 7.5 folds in CS relative to control. This cardiomyocyte population contraction is corroborated by histological reports of cardiomyocyte degeneration in CS patients since 1,980 (55). The upregulation of cardiac processes by CS cardiomyocytes might provide potential mechanistic explanation of the clinical symptoms reported by CS patients, such as atrial arrhythmias, syncope, palpitations, and fatigue. Regardless, mechanistic studies investigating cardiomyocyte pathobiology in CS are lacking and these findings that CS cardiomyocytes appear to exhibit upregulated immune activation and cardiac stress profiles relative to control exemplify the need to dedicate more efforts to understanding this immunologically complex disease.

In conclusion, our transcriptomic analysis reveals that, despite tissue-specific differences, both sarcoidotic T cells and macrophages exhibit attenuated activation profiles relative to control. Intriguingly, we show that both CS and PS T cells exhibit expansion of Th17.1 populations relative to control. In addition to our findings that CS cardiac fibroblasts exhibit upregulated pro-fibrotic and pro-inflammatory phenotypes, previously reported in other forms of sarcoidosis, we report autophagy upregulation as well. Our findings also revealed that CS cardiac endothelial cells exhibit upregulated pro-angiogenic and pro-inflammatory pathways. In addition to these canonical observations, our findings revealed that CS cardiac endothelial cells exhibit upregulated tissue remodeling but downregulated autophagy phenotypes as well. Lastly, our findings revealed that CS cardiomyocytes exhibit upregulated pro-inflammatory and cardiac stress profiles. While these findings provide more insights into the intricate nature of CS pathology, BAL is a poor representative of pulmonary pathologies involving an array of immune and stromal populations due to its myeloid bias. Moreover, as the authors behind the study that made the CS snRNA-Seq dataset publicly available did not publish certain clinical details about the CS patients whose sequencing data was utilized for this analysis, such as their stage of disease progression and treatment regimen, it is challenging to ascertain how our conclusions are generalizable regardless of disease stage and treatment. As the cardiac samples utilized for the CS analysis were explanted transplant specimens, it is reasonable to assume that CS donors recruited for this dataset had reached the chronic stage of their disease by the time of sample collection. Therefore, work involving transcriptomic analysis of PS pulmonary specimens and a more representative CS cohort is needed to better profile potentially pathological transcriptional phenotypes involved in PS and CS pathology. Furthermore, as this analysis focuses on transcriptional profiling of CS and PS, it is critical to conduct protein level studies as well as biomarker and cardiac function testing using sarcoidosis CS and pulmonary sarcoidosis samples in order to better elucidate the causative processes involved in sarcoidosis pathology regardless of tissue presentation.

## Data Availability

The original contributions presented in the study are included in the article/**Supplementary Material**, further inquiries can be directed to the corresponding author.

## Supplementary material

SUPPLEMENTARY FIGURE S1

Overview of the sn/scRNA-Seq workflow. Publicly available healthy & sarcoidotic sn/scRNA-Seq datasets were integrated separately for the heart & BALF comparisons. Following cell type clustering, cell type proportion, cell-cell communication and transcription factor analyses were conducted. Figure generated using Biorender.com.

SUPPLEMENTARY FIGURE S2

(**A**) UMAP clustering of integrated control and CS snRNA-Seq datasets by disease status. Metrics utilized for quality control are visualized on the right using violin plots that are stratified by disease status. (**B**) UMAP clustering of integrated control and PS scRNA-Seq datasets by disease status. Metrics utilized for quality control are visualized on the right using violin plots that are stratified by disease status. (**C**) Heatmaps showing the scaled expression of the top 10 markers of each identified biological cluster. (**D**) Pseudotime trajectory analysis conducted on cardiac stromal cells. Different parts of trajectory are highlighted and stratified by disease status. Pseudotime score indicates differentiation status.

SUPPLEMENTARY FIGURE S3

(**A**) Table showing number of Th17.1 cells in the heart and BAL stratified by disease status (top) and percentage of Th17.1 cells out of total T cells in the heart and BAL stratified by disease status. P-values are displayed above each table to indicate the corresponding statistical significance of Th17.1 changes in the heart across disease status. (**B**) Dot plot showing the cardiac gene expression of *TBX21* and *AOAH* in Th17.1 cells relative to other T cells. Average gene expression was scaled from 0 to 0.6. (**C**) Plot showing the ROC analysis results conducted in cardiac Th17.1 relative to other cardiac T cells. The AUC score is shown on the y-axis while the average log2(fold change) is shown on the x-axis. The closer a gene’s AUC score is to 1 the stronger its potential is to be a positive classifier while the closer it is to 0.5, the weaker its potential is to be a classifier. (**D**) Dot plots showing the expression of sarcoidosis-specific transcription factors (TFs) in the cardiac and BAL datasets in control and sarcoidosis MФ subpopulations. Average gene expression was scaled from 0 to 0.6.

SUPPLEMENTARY FIGURE S4

(**A**) Heatmap showing the scaled expression of the top 10 markers of each identified macrophage subcluster. (**B**) Dot plot showing the expression of markers used to identify each macrophage subcluster in the heart. Average gene expression was scaled from 0 to 2. (**C**) Top cell-cell communication pathways shown to be differentially upregulated in sarcoidotic BAL macrophages relative to control. The column graph shows the relative contribution of the highlighted pathways while the heatmaps show the direction of communication between the BAL macrophage clusters for each highlighted communication pathway. (**D**) Violin plot showing the gene expression of *NR1H3* in cardiac and BAL GPNMB+ macrophages stratified by disease status. (**E**) Dot plots showing the expression of sarcoidosis-specific transcription factors (TFs) in the cardiac and BAL datasets in control and sarcoidosis MФ subpopulations. Average gene expression was scaled from 0 to 0.6.

SUPPLEMENTARY FIGURE S5

(**A**) Heatmap showing the scaled expression of the top 10 markers of each identified fibroblast subcluster. (**B**) Dot plot showing the expression of markers used to identify each fibroblast subcluster in the heart. Average gene expression was scaled from 0 to 2. (**C**) Violin plots showing the expression of sarcoidosis-specific transcription factors (TFs) in control and sarcoidotic cardiac fibroblast subpopulations. The classification group of each TF is shown colored above its respective gene expression plot.

SUPPLEMENTARY FIGURE S6

(**A**) Heatmap showing the scaled expression of the top 10 markers of each identified endothelial cell subcluster. (**B**) Dot plot showing the expression of markers used to identify each endothelial cell subcluster in the heart. Average gene expression was scaled from 0 to 2. (**C**) Cell-cell communication tree of the CDH5 communication pathway between cardiac endothelial cell subpopulations. (**D**) Cell-cell communication tree of the PTPRM communication pathway between cardiac endothelial cell subpopulations.

SUPPLEMENTARY FIGURE S7

(**A**) UMAP clustering of cardiomyocyte subclusters by disease status. (**B**) Heatmap showing the scaled expression of the top 10 markers of each identified cardiomyocyte subcluster. (**C**) Feature plot showing the expression of *TNNI3* in the different cardiomyocyte subclusters.
