# Supplementary figures and images for: Integrative single-cell analysis of cardiac and pulmonary sarcoidosis using publicly available cardiac and bronchoalveolar lavage fluid sequencing datasets
Source: Front Cardiovasc Med. 2023 Jul 28;10:1227818. doi: 10.3389/fcvm.2023.1227818 (PMC10419306; doi:10.3389/fcvm.2023.1227818)

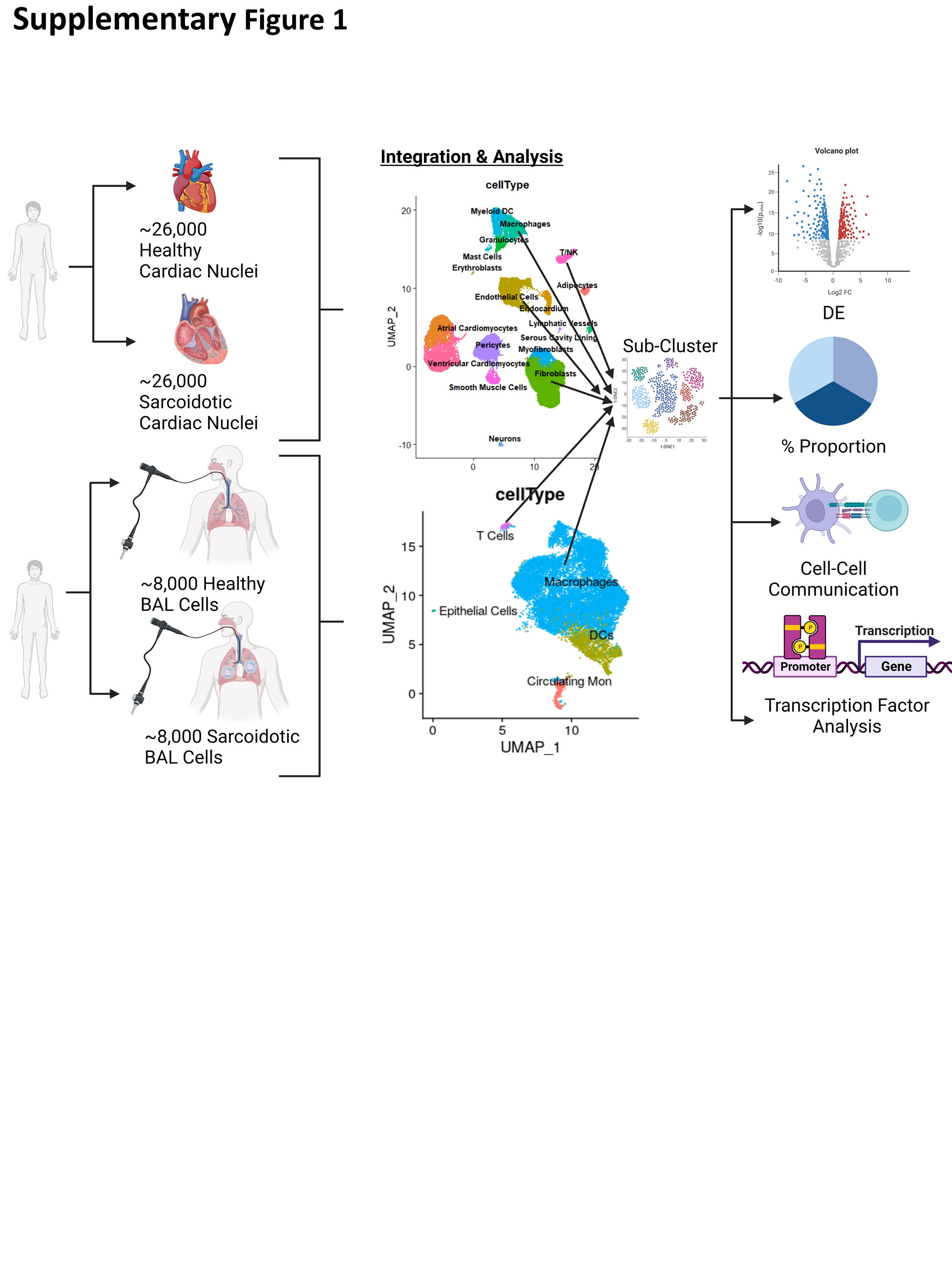

Supplement: Supplementary file 2 [file Image1.tif]

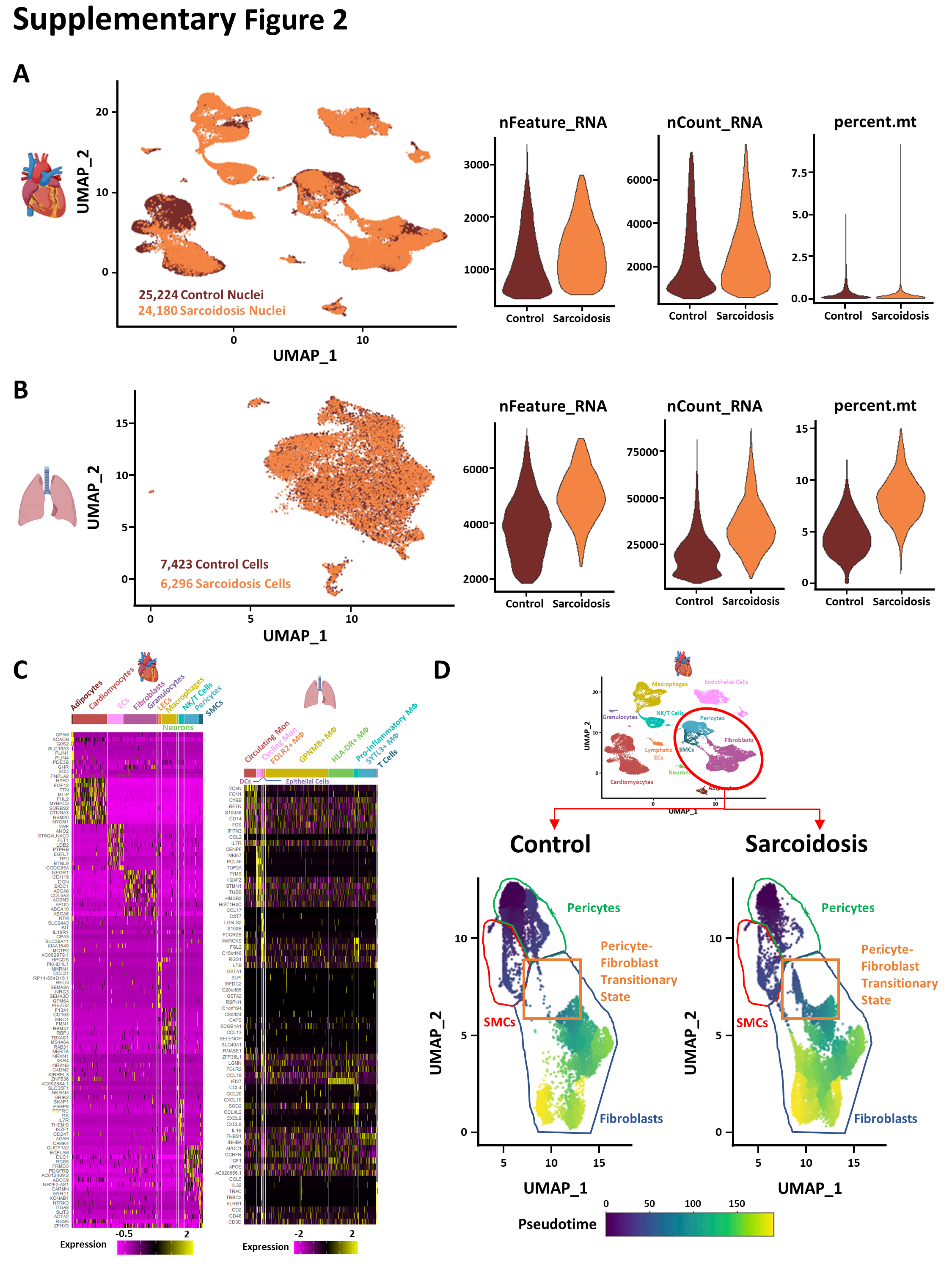

Supplement: Supplementary file 3 [file Image2.tif]

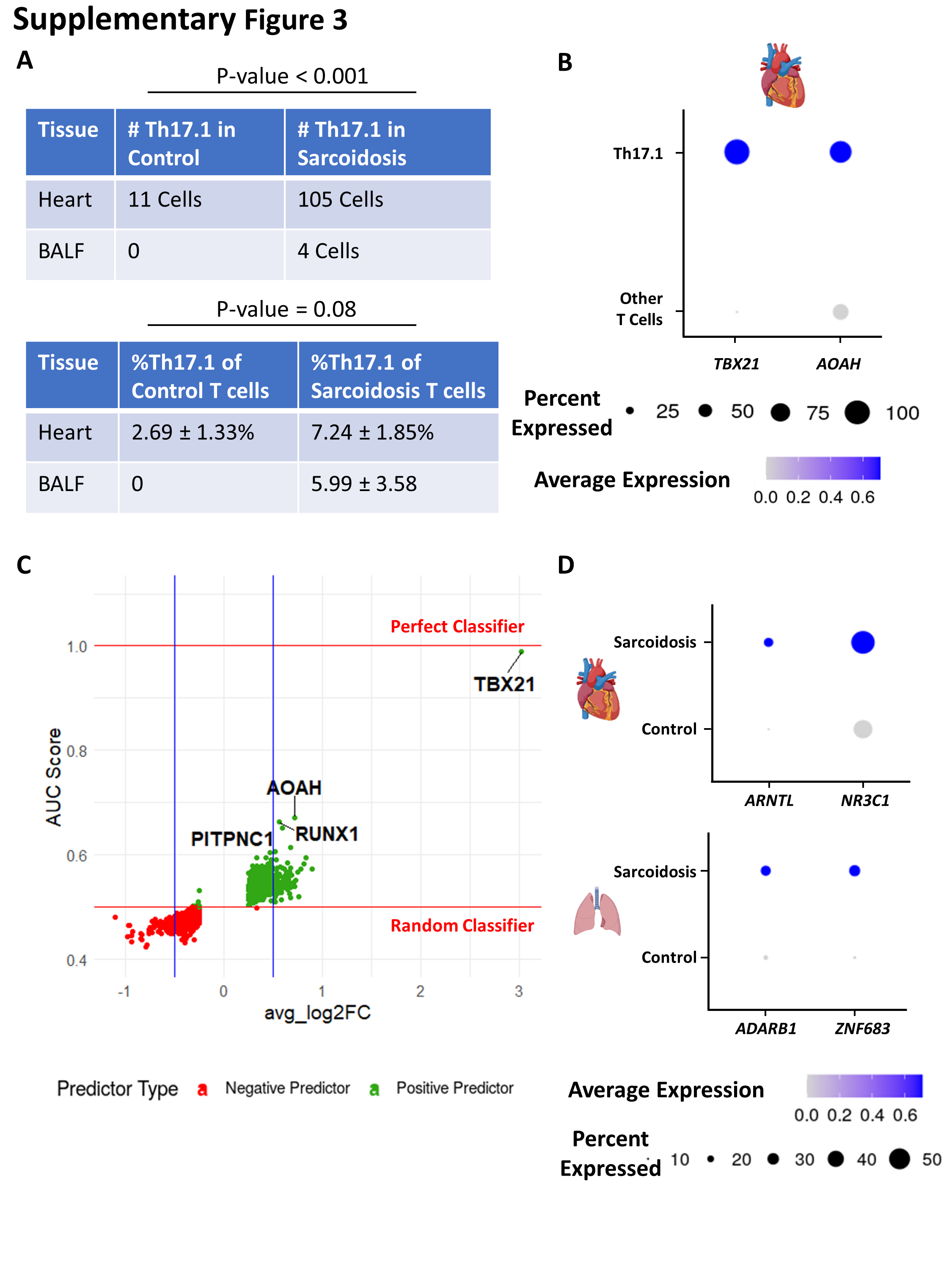

Supplement: Supplementary file 4 [file Image3.tif]

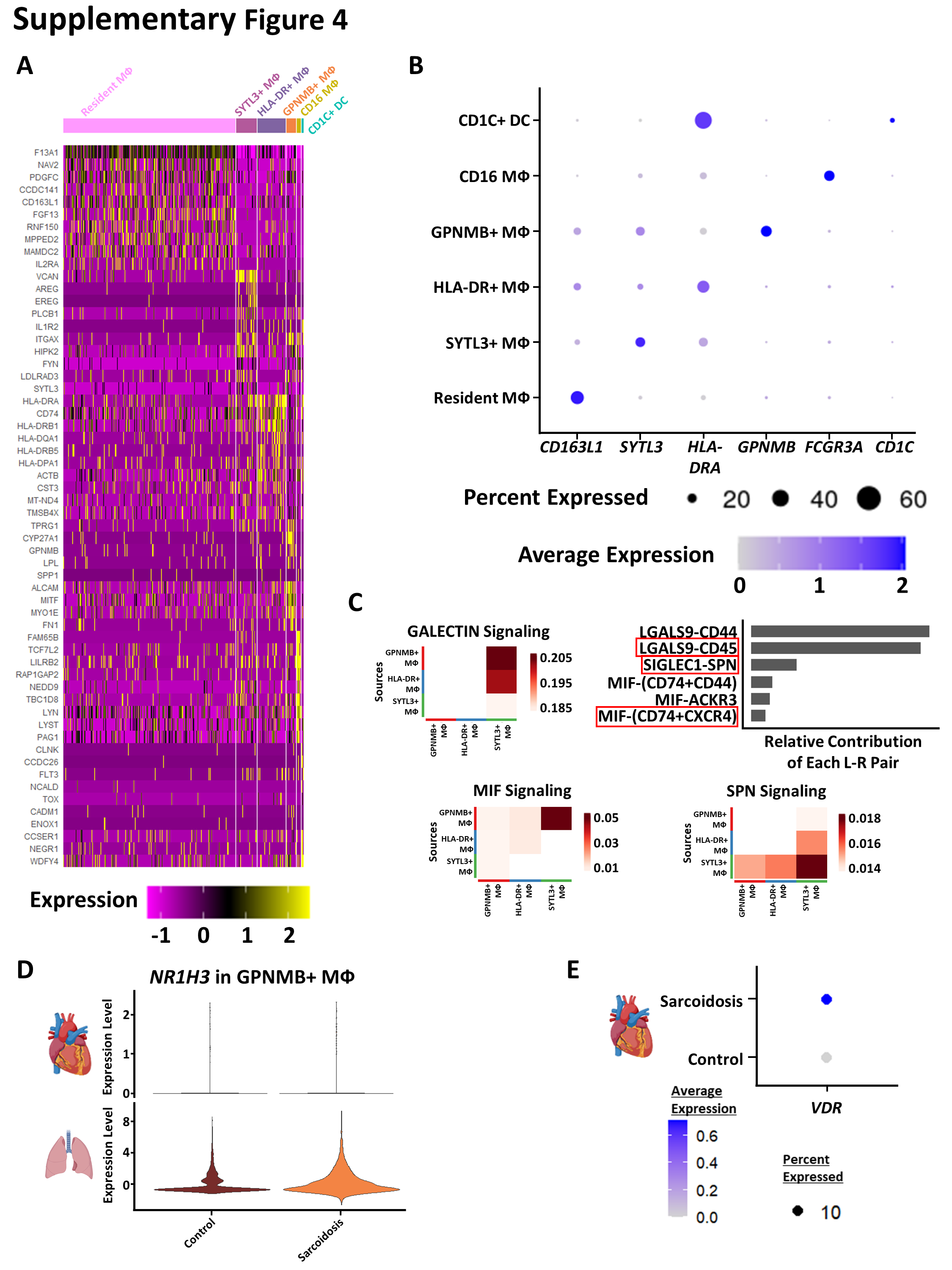

Supplement: Supplementary file 5 [file Image4.tif]

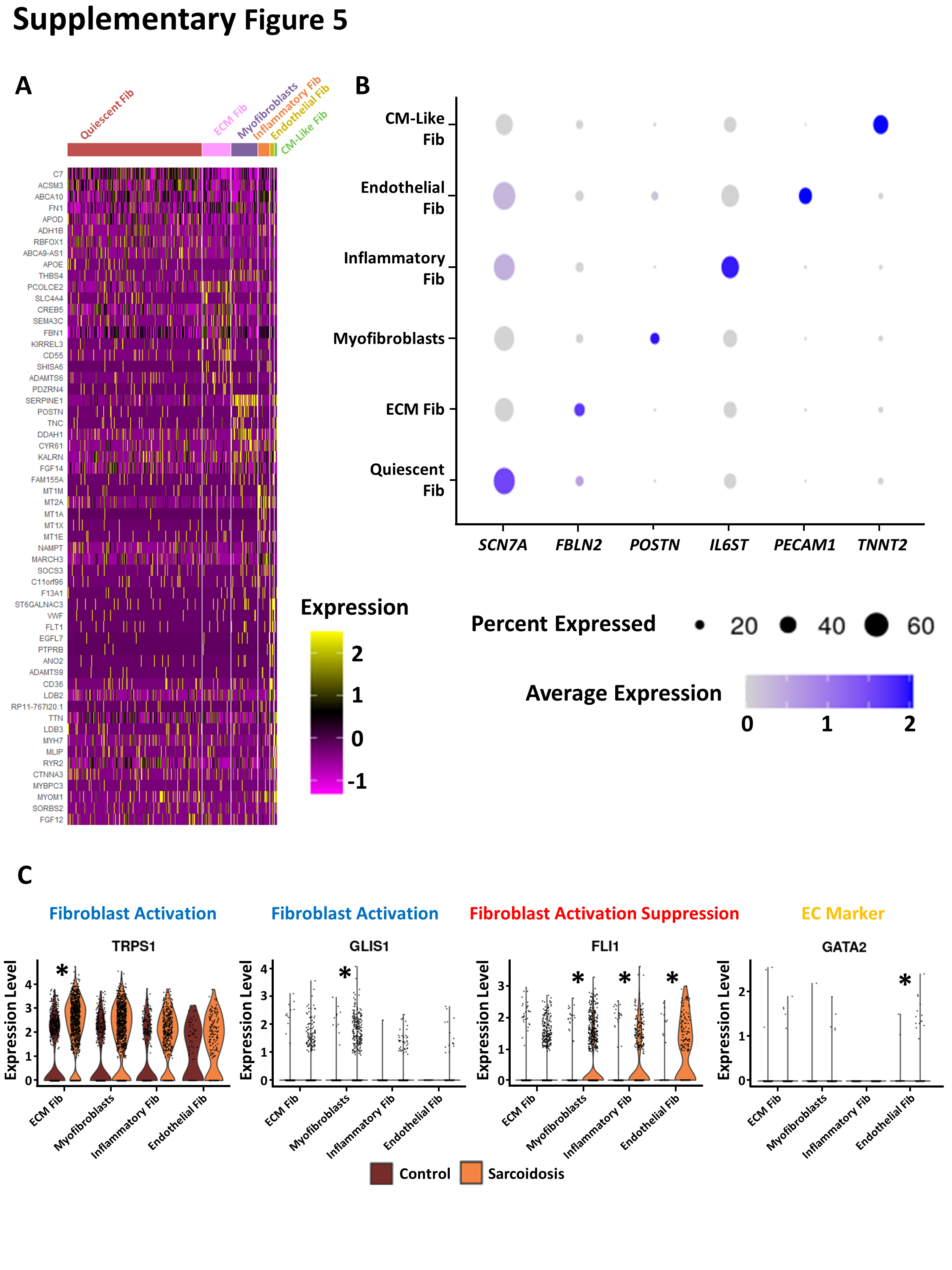

Supplement: Supplementary file 6 [file Image5.tif]

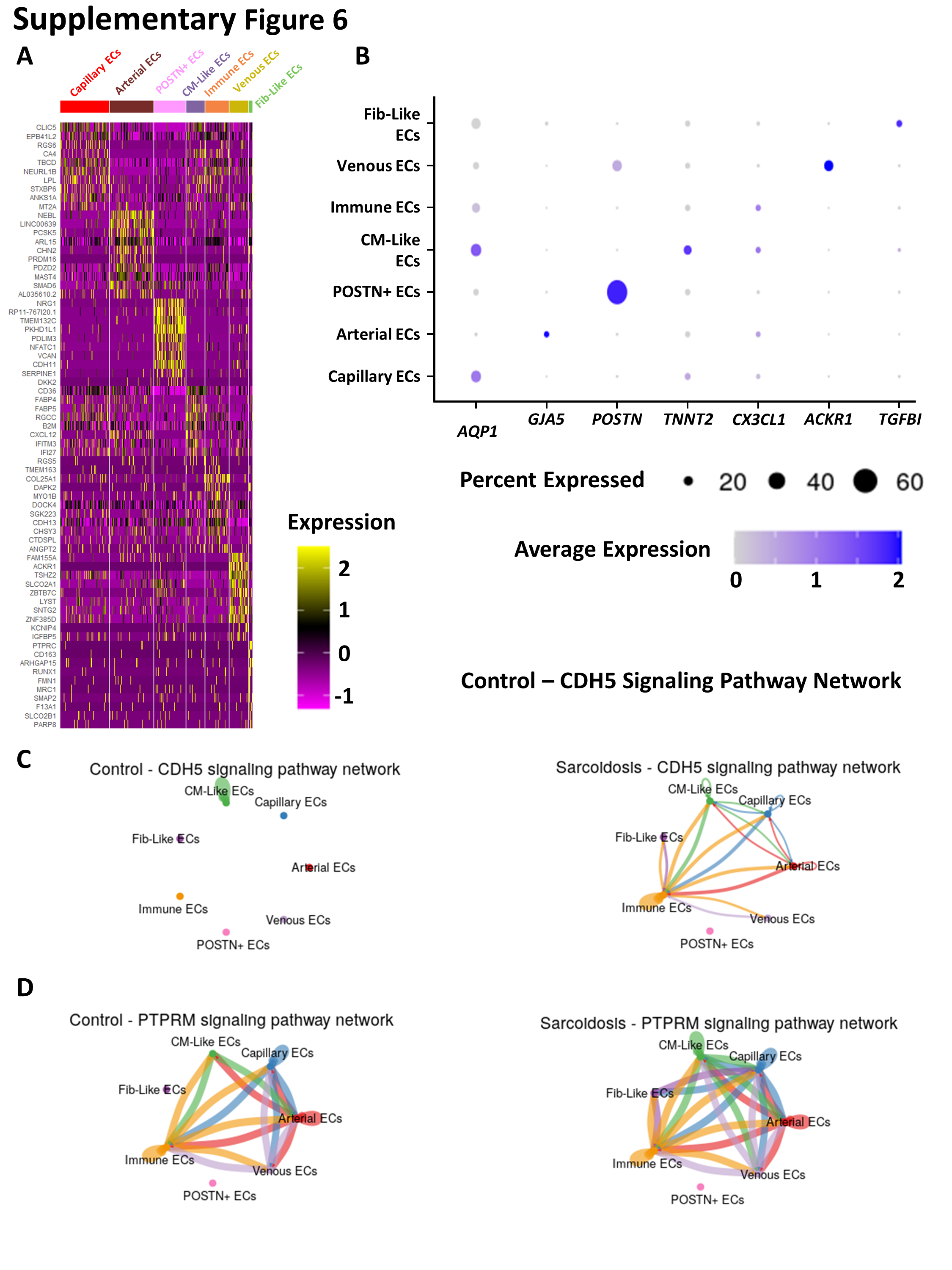

Supplement: Supplementary file 7 [file Image6.tif]

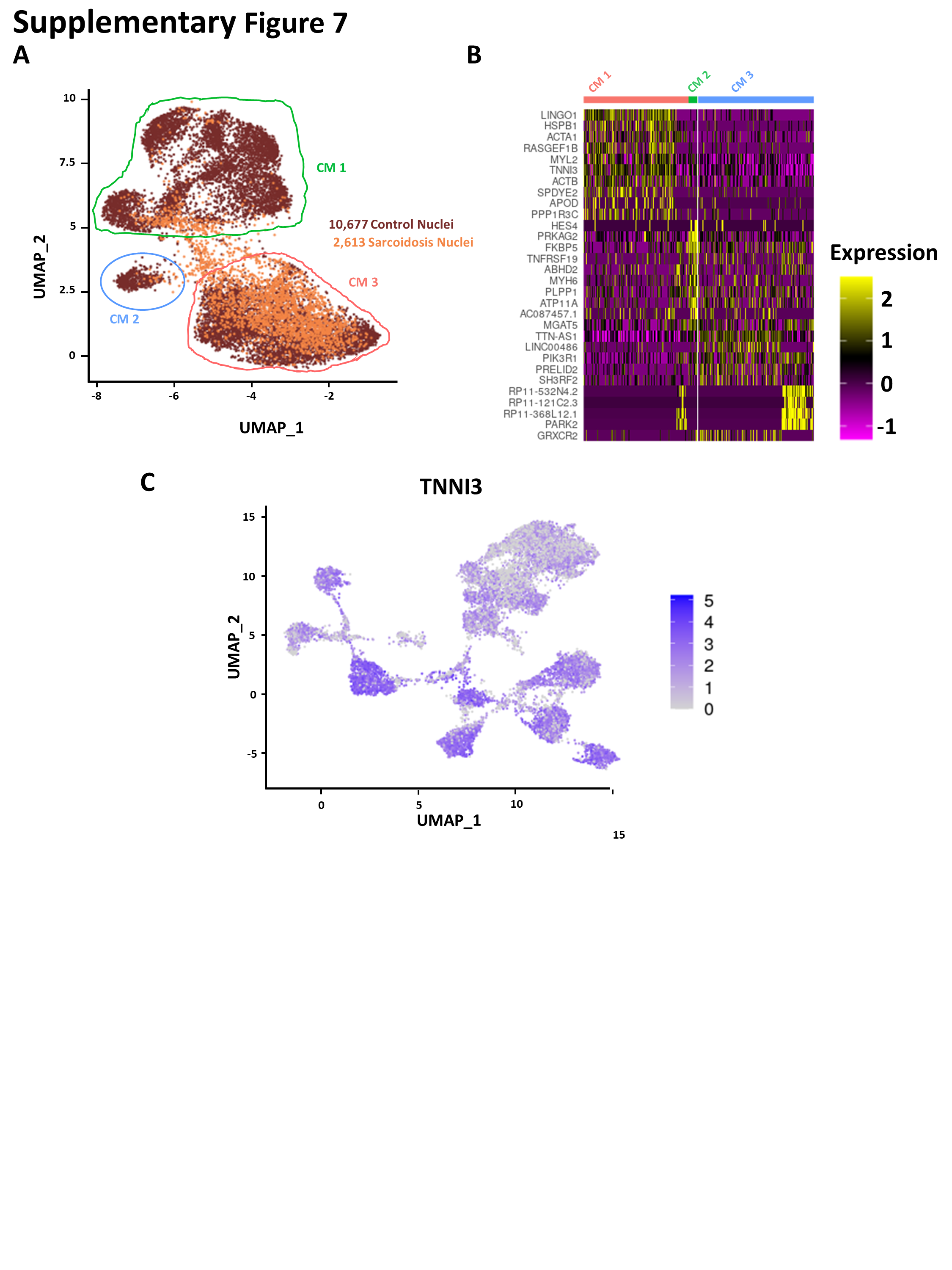

Supplement: Supplementary file 8 [file Image7.tif]
